# Reliability of patient-reported complications following hip or knee arthroplasty procedures

**DOI:** 10.1186/s12874-018-0645-0

**Published:** 2019-01-11

**Authors:** Sung Mu Heo, Justine M. Naylor, Ian A. Harris, Timothy R. Churches

**Affiliations:** 1 South Western Sydney Clinical School, UNSW Sydney, Level 2, Clinical Building, Liverpool Hospital, Cnr Elizabeth and Goulburn Sts, Liverpool NSW, 2170 Australia; 2 Ingham Institute for Applied Medical Research, 1 Campbell St, Liverpool NSW, 2170 Australia; 3 Whitlam Orthopaedics Research Centre, Level 2, 1 Campbell St, Liverpool NSWl, 2170 Australia

**Keywords:** Knee arthroplasty, Hip arthroplasty, Patient-reported outcome measures, Complications, Joint replacement registry, Validation study

## Abstract

**Background:**

Patient reported outcomes are increasingly used to assess the success of surgical procedures. Patient reported complications are often included as an outcome. However, these data must be validated to be accurate and useful in clinical practice.

**Methods:**

This was a retrospective descriptive study of 364 patients who had completed their six-month follow-up review questionnaire in the Arthroplasty Clinical Outcomes Registry, National (ACORN), an Australian orthopaedic registry. Patient-reported complications following total hip arthroplasty (THA) and total knee arthroplasty (TKA) were compared to surgeon-reported complications recorded in their electronic medical records at their various follow-up appointments. Sensitivity, specificity, positive predictive value and negative predictive value were calculated. Agreement was assessed using percentage agreement and Cohen’s kappa.

**Results:**

Patient-reported data from the ACORN registry returned overall low sensitivity (0.14), negative predictive value (0.13) and kappa values (0.11), but very high specificity (0.98), positive predictive value (0.98) and agreement values (96.3%) for reporting of complications when compared to surgeon-reported data. Values varied depending on the type and category of complication.

**Conclusion:**

Patients are accurate in reporting the absence of complications, but not the presence. Sensitivity of patient-reported complications needs to be improved. Greater attention to the clarity of the questions asked may help in this respect.

**Electronic supplementary material:**

The online version of this article (10.1186/s12874-018-0645-0) contains supplementary material, which is available to authorized users.

## Background

Patient reported outcome measures (PROMs) are a reliable tool for understanding patients’ perceived outcomes, and are typically implemented via telephone or mail follow-up via a centralised registry [1]. Complications are often included in such registries as these data can be analysed to inform best practice and reduce the rates of these complications and their associated economic and social burden [2–5].

The Arthroplasty Clinical Outcomes Registry, National (ACORN) is an Australian orthopaedic registry collecting clinical and patient-reported outcomes by telephone interview following elective primary and revision total hip arthroplasty (THA) and total knee arthroplasty (TKA) at six months (± one month) post-operatively. The ACORN 2016 annual report reported post-discharge complication rates of 0.055 to 5.4% for THA and 0.0 to 13.0% for TKA [6]. However, to utilise these data to understand complication rates following surgery and influence current practice, the accuracy of the data must be assessed.

Previous studies have compared patient-reported complications (PRC) to clinical examination and medical records in general, inguinal hernia repair, bone marrow transplant, varicose vein, spinal, prostate, gynaecological oncology and orthopaedic surgery [7–19]. Overall, these studies have indicated high negative predictive values (NPV, 95.0 to 98.2%) but low positive predictive values (PPV, 26.0 to 83.3%) and varying levels of concordance (56.4 to 97.2%) and agreement (11.0 to 100.0%). One study on bone marrow transplant patients showed varying sensitivity (52.9 to 100.0%) and specificity (75.4 to 100.0%) dependent on complication type [13]. Another study monitoring patients’ ability to report surgical site infections reported high sensitivity (83.3%) and specificity (93.7 to 98.1%) [16]. Sensitivity refers to patients’ ability to report the true presence of complications, whilst specificity refers to their ability to report the true absence of complications, when compared to health professionals. PPV and NPV refers to the likelihood of a patient’s positive or negative reporting of complications being correct. Of the three orthopaedic-specific studies, concordance was high for well-defined complications such as deep vein thrombosis and pulmonary embolism, but low for more ambiguous complications such as major bleeding and numbness [17–19].

This study aims to assess the reliability of PRC following THA and TKA recorded in ACORN compared to surgeons’ medical records by calculating sensitivity, specificity, PPV, NPV and agreement values.

## Methods

### Patient population

Patients were included in this study if they had completed a six-month post-operative follow-up interview with ACORN. Patients were included in the ACORN registry if the person was 18 years of age or over, their arthroplasty procedure (primary or revision) of the hip or knee was elective, the surgery was undertaken at a hospital participating in the registry, and the person was not cognitively impaired or unable to consent to participation. Approximately fifty patients who had most recently completed the six-month post-operative follow-up were randomly chosen from each of the six surgeons with the highest volume of procedures captured in ACORN in 2015.

This study was approved by the Hunter New England Human Research Ethics Committee (HREC) as an incorporated sub-study to ACORN. ACORN utilises a verbal opt-out consent process, informed by an HREC-approved written patient information sheet provided to and discussed with each patient at their pre-operative clinical assessment. The consent provides for patient data to be included in the ACORN registry and used for post-operative follow-up of their complications and outcomes via a telephone questionnaire, for the purposes of quality assurance and research [6].

### Sample size calculation

Sample size calculation for statistical power to detect a Cohen’s *kappa* agreement statistic in the range 0.4 to 0.7 with a mean complication prevalence of 10% (range 1.0 to 15.0%) and with a standard *α* parameter of 0.05, was undertaken using the *kappaSize* package for R, yielding a minimum sample size of 300 patients [20]. To obtain an even distribution of patients, data from at least 50 patients from each surgeon who had most recently completed their six-month follow-up at April 2016 were acquired. An R script was used to randomly sample from all patients for each surgeon from 2015 from January to October inclusive, and the search was extended backward into 2014 if the patient volume was insufficient for some surgeons.

### ACORN six-month follow-up data collection form

The full ACORN six-month follow-up data collection form includes several subjective questions regarding satisfaction, perceived success, EuroQol 5 Dimensions - 5 Level (EQ5D-5L) and EuroQol-Visual Analogue Scale (EQ-VAS) scores, and Oxford Hip or Knee Scores [6]. Complications are also captured as a part of the registry data.

The questions regarding complications were grouped into readmission, reoperation and other complications. Response options to readmission and reoperation were *Yes/No/Unstated or unknown*. If patients responded *Yes* for readmission, they were asked about primary reason and hospital of admission. If patients responded *Yes* for reoperation, they were asked to state the reason for reoperation.

If patients responded *Yes* to complications not requiring readmission, patients were further asked to specify their complication with an open ended question as to not prompt the patient. Callers then recorded the complications on the standard data collection form which consists of the following; surgical site infection (SSI) requiring oral antibiotics, SSI requiring intravenous antibiotics, deep vein thrombosis (DVT) index leg, DVT other leg, DVT both legs, pulmonary embolism (PE), dislocation, joint stiffness, bladder infection or urinary retention, fracture, unexpected pain, cardiac, stroke, leg length discrepancy, joint or lower limb swelling, paraesthesia or numbness, cellulitis, neuropathy, muscle weakness, respiratory infection, other, and unknown.

Readmission, reoperation and twenty-two separate complications were considered in this study. Twenty of these complications (excluding *other* and *unknown or not stated*) were arranged into groups based on similarity, in order to additionally assess validity and agreement within broader categories, as shown in Table 1.

**Table 1:** Complications grouped by category used for analysis

Category	Included complications
Thromboembolic events	DVT index leg, DVT other leg, DVT both legs, Pulmonary embolism
Infections	SSI requiring oral antibiotics, SSI requiring IV antibiotics, cellulitis
Problems involving the joint	Joint stiffness, Fracture, Leg length discrepancy, Joint or lower leg swelling, Dislocation
Medical complications	Bladder infection or retention, Cardiac, Stroke, Neuropathy, Respiratory infection
Subjective complications	Unexpected pain, Paraesthesia or numbness, Muscle weakness

### Data abstraction

Information on post-discharge complications for each patient was abstracted by the lead author from the electronic medical records maintained by each surgeon in their practice. Abstraction was repeated for a subset to confirm the reliability of the data collection. The items abstracted were the same as those collected from each patient at the six-month post-operative follow-up interview using the identical questionnaire used by ACORN. Patients without record of follow-up review by surgeons were excluded and a substitute patient from the randomised list of patients was substituted. Data collection continued until at least 50 patients with follow-up had been collected for each surgeon. Where more time was available, data from additional randomly-chosen patient records were collected from each surgeons’ records.

To avoid possible bias, the investigator abstracting data from surgeons’ private practices was blinded to the results of the six-month follow-up interview for the selected patients from the ACORN database. Following the completion of data abstraction from surgeons’ rooms and recording results into a database, these records were locked and unable to be changed.

### Statistical analysis

The data were analysed by calculating the sensitivity, specificity, PPV, NPV, percentage agreement and unweighted Cohen’s *kappa* coefficient. Cohen’s *kappa* is a measurement of inter-rater reliability, which adjusts for chance agreement between raters, and is usually interpreted categorically: values less than or equal to zero denote no agreement; 0.01-0.20 slight agreement; 0.21-0.40 fair agreement; 0.41-0.60 moderate agreement; 0.61-.80 substantial agreement, and; 0.81-1.00 almost perfect agreement. The surgeons’ medical records were treated as the gold standard in this study.

To investigate whether additional factors influence these classification and agreement metrics, analyses were also performed with patients categorised by surgeon, the joint operated on, and the time between surgery and follow-up review (which is not always at six months for surgeon follow-up, unlike the ACORN follow-up). The surgeon follow-up times were categorised into <6 weeks, 6–8 weeks, 3–5 months, 6 months and 6–12 months, and were individually compared to the 6-month ACORN data. All analyses were completed using the R statistical computing software environment for statistical computing version 3.3.3 [21, 22] (Additional file 1).

## Results

In the random sampling process, 364 patients were selected, of whom 340 had at least one recorded review with a surgeon within six months of surgery. Overall, there were more females than males, and more TKA than THA. No significant between-patient differences in characteristics were observed between surgeons, apart from a systematic difference in follow-up time, which was driven by individual surgeon’s usual practice. Surgeons A, B and E reviewed the bulk of their patients within 8 weeks, whereas surgeons C, D and F reviewed closer to the six-month mark.

Table 2 summarises the characteristics of selected patients and time between follow-up consultations, compared by surgeon.

**Table 2:** Summary of patient characteristics

	Surgeon						
	A	B	C	D	E	F	All
Persons	63	73	51	53	48	52	340
Males	20	26	19	16	17	20	118
Females	43	47	32	37	31	32	222
Mean age (years)	68	67	67	69	72	68	68
StdDev	8.1	12.8	9.6	9.1	8.1	10.4	10.1
Joint (n)							
Hips	11	25	21	16	13	16	102
Knees	52	48	30	37	35	36	238
Follow-up time (n)							
<6 weeks	15	1	0	0	7	0	23
6–8 weeks	36	38	9	6	17	15	121
3–5 months	4	7	3	14	6	6	40
6 months	4	20	38	28	12	27	129
>12 months	4	7	1	5	5	4	26

A total of 163 complications were reported by 77 patients. The results of the complete analysis are summarised in Table 3.

**Table 3:** Validity and agreement values for PRC when compared to surgeons’ notes

Complication	*n* ^a^	TT^b^	TF^c^	FT^d^	FF^e^	Sens	Spec	PPV	NPV	Agree %	*kappa*
Re-admission	340	9	9	7	315	0.50	0.98	0.56	0.97	95.29	0.50
Re-operation	340	3	2	2	333	0.60	0.99	0.60	0.99	98.82	0.59
Thromboembolic event	1360	1	3	4	1352	0.25	1.00	0.20	1.00	99.49	0.22
DVT index leg	340	1	2	3	334	0.33	0.99	0.25	0.99	98.53	0.28
DVT other leg	340	0	0	0	340	NA	1.00	NA	NA	100.00	1.00
DVT both legs	340	0	0	0	340	NA	1.00	NA	NA	100.53	1.00
Pulmonary embolism	340	0	1	1	338	0.00	1.00	0.00	1.00	99.41	0.00
Infections	1020	2	13	5	1000	0.13	1.00	0.29	0.99	98.24	0.17
SSI requiring oral AB	340	2	9	5	324	0.18	0.98	0.29	0.97	95.88	0.20
SSI requiring IV AB	340	0	0	0	340	NA	1.00	NA	NA	100.00	1.00
Cellulitis	340	0	4	0	336	0.00	1.00	NA	0.99	98.82	0.00
Joint problems	1700	6	31	66	1597	0.16	0.96	0.08	0.98	94.29	0.08
Dislocation	340	0	0	0	340	NA	1.00	NA	NA	100.00	1.00
Stiffness	340	0	6	28	306	0.00	0.92	0.00	0.99	90.00	-0.03
Fracture	340	0	0	1	339	NA	1.00	NA	NA	99.71	0.00
Length discrepancy	340	1	9	4	326	0.10	0.99	0.20	0.97	96.18	0.12
Swelling	340	5	16	33	286	0.24	0.90	0.13	0.95	85.59	0.10
Medical complications	1700	1	11	5	1683	0.08	1.00	0.17	0.99	99.06	0.11
Respiratory infection	340	0	0	0	340	NA	1.00	NA	NA	100.00	1.00
Cardiac	340	0	0	1	339	NA	1.00	NA	NA	99.71	0.00
Stroke	340	0	0	0	340	NA	1.00	NA	NA	100.00	1.00
Urinary infection/retention	340	1	2	1	336	0.33	1.00	0.50	0.99	99.12	0.40
Neuropathy	340	0	9	3	328	0.00	0.99	0.00	0.97	96.46	-0.01
Subjective complications	1020	11	67	55	887	0.14	0.94	0.17	0.93	88.04	0.09
Unexpected pain	340	10	58	16	256	0.15	0.94	0.38	0.82	78.24	0.11
Paraesthesia/numbness	340	1	7	28	304	0.12	0.92	0.03	0.98	89.71	0.02
Muscle weakness	340	0	2	11	327	0.00	0.97	0.00	0.99	96.18	-0.01
Other complications	340	0	9	7	324	0.00	0.98	0.00	0.97	95.29	-0.02
Unknown	340	0	0	0	340	NA	1.00	NA	NA	100.00	1.00
All complications^f^	7480	21	134	142	7183	0.14	0.98	0.13	0.98	96.31	0.11

^a^The denominator for the combined complications is the number of subjects (340) multiplied by the number of individual complications in the combined category

^b^TT = True positives: both surgeons reported the complication

^c^TF = False negatives: surgeons reported the complication, patients did not

^d^FT = False positives: surgeons did not report the complication, but patients did

^e^FF = True negatives: neither surgeons nor patients reported the complication

^f^Overall complications exclude re-admission and re-operation

The proportions of positive agreements (true/true, denoted TT) were low across all complications, with the highest rates observed for readmission and unexpected pain. The highest rates of FT (surgeon/patient) disagreement were in stiffness, lower limb swelling and paresthesia, whilst highest rates of TF (surgeon/patient) disagreement were in unexpected pain. There were high negative agreements (FF) rates throughout all complications due to the low prevalence of most complications. As a result, low values for sensitivities and PPV and high values for specificities and NPV were observed.

With the exception of readmission and reoperation, patients’ sensitivity did not exceed 0.33 when compared to surgeons. Overall sensitivity was 0.14, with nine complications having no result due to the absence of true positives and false negatives, and six complications having zero sensitivity due to an absence of true positives. PPV were similar, with a highest value of 0.38, and 0.13 overall. In contrast, the lowest specificity value was 0.92, and 0.98 overall. NPV was equally high at 0.98 overall and all values were greater than 0.90, with the exception of unexpected pain (0.82).

Twenty-one of 24 complication types showed greater than 90% agreement and agreement was 96.31% overall. In terms of the *kappa* statistic, two complications were in fair agreement, five in slight agreement and eight showed no agreement. Aside from urinary infection (0.40), values were lower than or equal to 0.28, and an overall *kappa* value of 0.11 was observed. Patients and surgeons unanimously reported no complications for seven complications, yielding 100% agreement and a *kappa* of 1.00.

Table 4 provides subgroup analyses by surgeon, joint and follow-up time.

**Table 4:** Validity and agreement values for PRC when compared with surgeons’ notes, categorised by surgeon, joint and follow-up time

Complication	*n* ^a^	TT^b^	TF^c^	FT^d^	FF^e^	Sens	Spec	PPV	NPV	Agree %	*kappa*
Surgeon											
Surgeon A	1386	3	12	39	1332	0.20	0.97	0.07	0.99	96.32	0.09
Surgeon B	1606	6	16	32	1552	0.27	0.98	0.16	0.99	97.01	0.19
Surgeon C	1122	3	16	17	1086	0.16	0.98	0.15	0.99	97.06	0.14
Surgeon D	1166	4	52	11	1099	0.07	0.99	0.27	0.95	94.60	0.09
Surgeon E	1056	1	13	26	1016	0.07	0.98	0.04	0.99	96.31	0.03
Surgeon F	1144	4	25	17	1098	0.14	0.98	0.19	0.98	96.33	0.14
Joint											
Hip	2244	5	46	16	2177	0.10	0.99	0.24	0.98	97.24	0.13
Knee	5236	16	88	126	5006	0.15	0.98	0.11	0.98	95.91	0.11
Follow-up time											
<6 weeks	506	1	1	15	489	0.50	0.97	0.06	0.99	96.84	0.10
6–8 weeks	2662	3	19	44	2596	0.14	0.98	0.06	0.99	97.63	0.08
3–5 months	880	2	18	21	839	0.01	0.98	0.09	0.98	95.57	0.07
6 months	2838	13	82	50	2693	0.14	0.98	0.21	0.97	95.35	0.14
6–12 months	572	2	14	12	544	0.12	0.98	0.14	0.97	95.45	0.11

^a^The denominator for the combined complications is the number of subjects in each subgroup multiplied by the number of individual complications (22) assessed for each subgroup

^b^TT = True positives: both surgeons reported the complication

^c^TF = False negatives: surgeons reported the complication, patients did not

^d^FT = False positives: surgeons did not report the complication, but patients did

^e^FF = True negatives: neither surgeons nor patients reported the complication

## Discussion

Orthopaedic clinical registries are becoming increasingly popular due to their ability to monitor results of surgery in a time- and cost-efficient manner, whilst incorporating the patient’s perspective in the assessment of their surgery. To use this information to influence current practice, however, the accuracy of these data must be assessed.

This study has demonstrated that when patient-reported complication data from a clinical registry is assessed against surgeon notes, they show high specificity, NPV and percentage agreement. These indicate that patients are able to accurately report that they did not experience any complications, as seen in the high true negative results. Since the rates of complications following THA and TKA procedures are low, this study suggests that registries are adequately valid and reliable for assessing complication rates following TKA and THA procedures.

On the other hand, very low sensitivity and PPV were demonstrated, indicating that precise rates of specific complication types may not be adequately estimated from patient-reported data, a finding which is in concordance with previous studies. Three studies on patients identifying surgical site infections and one on hernia repair patients showed that PRC typically showed lower PPV and sensitivity than NPV and specificity [9, 15, 16]. Registries may be a better tool for assessing complications if these values could be improved, but this is challenging when complication rates are low, as small degrees of disagreement can have large effects on calculated sensitivity values and NPV, which in turn can be masked in the specificity and percentage agreement values due to the large number of true negative values (*kappa* coefficient is addressed in a later section). Nevertheless, if the true negative results were ignored, there were only 21 (7.1%) instances in this study of patients and surgeons agreeing on the presence and type of complications, in comparison to 276 (92.9%) instances of disagreement, out of a total of 297 comparisons. This indicates that, in the presence of complications, patients cannot reliably report occurence of complications.

Where patient and surgeon reports disagreed, patients were more likely to over-report complications in most categories. Of course, high rates of reporting by patients for stiffness, paraesthesia and muscle weakness may be reasonable, if we assume patients are more inconvenienced by minor complications than surgeons often believe. Patients were more likely to under-report leg length discrepancies and superficial infections. Patients with minor leg-length discrepancies may have few symptoms which may explain the under-reporting by patients, whereas surgeons place great importance on leg length due to its possible detrimental outcomes [23]. This study showed poor patient specificity in identifying SSI, a finding that was also observed in a study by Zellmer et al., who suggested that may be improved with validated infection education material [24].

The complications data for ACORN are collected using a *yes* or *no* answer. However, complications such as swelling and stiffness are somewhat expected events following surgery as part of a natural healing process. The time frame and degree of debility caused by these complications should be sought, rather than mere presence. Dushey et al. noted similar deficiencies in questionnaires and proposed that quantitative or degree of seriousness criteria should be added when enquiring after the less objective complications [17]. A similar study on general surgery procedures interviewed patients by asking if “an adverse outcome had occurred between discharge and 30 days after discharge”, and found that patients grossly over-reported complications as they would describe their symptoms (e.g. pain and fever) compared to surgeons who observed diagnoses (e.g. infection)[14]. Another study allowed patients to freely describe whether “any complications [arose] as a consequence of [their] operation three months ago?” They critiqued their own methodology, and concluded that clear definitions could improve concordance rates [11].

ACORN callers similarly enquire if the patients have experienced a complication and then ask the patient to specify details, without prompting for specific types of complication. Although this provides a window into patients’ experience and recollection of post-operative complications, it does not provide information on the extent or severity. For example, muscle weakness can refer to slight difficulty in movement to complete immobilisation. Clinical registries such as ACORN may benefit from further enquiring about severity of complications.

This study observed high rates of false negative results for unexpected pain as seen by Visser et al. and Franneby et al. [8, 14]. Joint pain is a major reason for patients undergoing THA and TKA, and hence, it was expected that patients would over-report pain if it continued following their procedures. This may be an incorrect assumption, and the observed results may actually be because the questionnaire refers to this specifically as “unexpected pain”, whilst surgeons (who do not follow a *pro forma* set of questions) may have noted any pain that the patient reported. This suggests that patients may in fact expect a certain amount of pain following surgery, and added measures of clarifying and quantifying these complications in questionnaires may help improve the accuracy of PRC.

The three existing orthopaedic studies seeking to validate PRC were limited as they only assessed the accuracy of patients who reported complications, and not the accuracy of those who reported no complications. This prevented them from measuring validity values as such as sensitivity and specificity, and they were prone to selection bias due to only investigating the group of patients who reported complications. Two of these studies [17, 19] referenced the same study by Parimi et al. [25], which reported false negative rates of 0.28% in patients reporting simply if they had had a THA, and hypothesised that similar false negative rates may occur with PRC. A strength of our study was that it investigated both groups, and was able to assess the ability of patients to accurately report when they did have a complication, as well as when they did not, and the findings of this study support the assumptions made by Dushey et al. and Greenbaum et al. that false negative rates are low.

Although Cohen’s *kappa* accounts for chance agreement, the literature has noted that the assumptions made about rater independence may overestimate chance agreement, thereby underestimating the agreement value [26, 27]. The implication for his study is that because of the low incidence of many of the complications, the *kappa* statistics are not robust to very small changes in agreement.

Patients followed up outside the six-month mark were not excluded because in clinical practice not all patients are reviewed at the same time point by surgeons. This was addressed by including subgroup analyses in which patients reviewed at six months post-surgery can be compared to those followed up at different times, which showed no significant differences. Further, this study accepted the surgeons’ records as gold standard as it reflects the real-life surgeon awareness of patient complications. Studies have discussed that although surgeons may have a better idea about what constitutes “true” medical and surgical complications, only the patients have the complete picture of adverse events [8, 11, 28]. Surgeons may also be susceptible to overlooking minor complications and keeping incomplete or inaccurate records, and alternative care sought from other health services for complications will not have been captured [14]. However, using patient recollection as the gold standard has its own set of problems, as it may be subject to recall bias. Both sources have limitations and this study suggests that agreement values (percentage agreement) may be more appropriate in assessing the accuracy of PRC.

## Conclusion

Accurate but efficient ascertainment of complication rates following surgery remains a highly important aspect of not only surgeon appraisal, but also of patient satisfaction and continuing improvements in medical care. The high concordance for true negative results along with high specificity, NPV and percentage agreement found in this study are encouraging, as it indicates that complication rates following THA and TKA are low, and PRC are accurate in this regard. However, the low sensitivity and PPV must be improved, and we suggest that improved wording and clarity of questionnaires used by registries to elicit these data from patients would aid in achieving this.

## Additional file

Additional file 1 Full report on study with additional tables and R code. (PDF 732 kb)

## Abbreviations

ACORN: Arthroplasty clinical outcomes registry, National
THA: Total hip arthroplasty
TKA: Total knee arthroplasty
PRC: Patient-reported complications
PPV: Positive predictive value
NPV: Negative predictive value
ACORN: Arthroplasty clinical outcomes registry, National
HREC: Human research ethics committee
EQ-5D-5L: EuroQol-5 Dimensions-5 level
EQ-VAS: EuroQol-visual analogue scale
SSI: Surgical site infection
DVT: Deep vein thrombosis
PE: Pulmonary embolism
TT: True positives (Surgeons reported, patients reported)
TF: False negatives (Surgeons reported, patients did not report)
FT: False positives (Surgeons did not report, patients reported)
FF: True negatives (Surgeons did not report, patients did not report)
AB: Antibiotic
IV: Intravenous

## References

[1] Siljander M, McQuivey K, Fahs A, Galasso L, Serdahely K, Karadsheh M (2018). Current trends in patient-reported outcomes measures in total joint arthroplasy: A study of 4 major orthopaedic journals. J Arthroplasty.

[2] Capozzi J, Rhodes R (2010). Examining the ethical implications of an orthopaedic joint registry. J Bone Joint Surg Am.

[3] Lacny S, Bohm E, Hawker G, Powell J, Marshall D (2016). Assessing the comparability of hip arthroplasty registries in order to improve the recording and monitoring of outcome. Bone Joint J.

[4] Bilimoria K, Liu Y, Paruch J, Zhou L, Kmiecik T, Ko C (2013). Development and evaluation of the universal ACS NSQIP surgical risk calculator: A decision aid and informed consent tool for patients and surgeons. J Am Coll Surg.

[5] Saarinen I, Malmivaara A, Miikki R, Kaipia A (2018). Systematic review of hospital-wide complication registries. BJS Open.

[6] Churches T, Naylor J, Harris I (2017). Arthroplasty Clinical Outcomes Registry National (ACORN) Annual Report 2016. Ingham Institute for Applied Medical Research, 1 Campbell St, Liverpool NSW 2170.

[7] Black N, Petticrew M, Ginzler M, Flood A, Smith J, Williams G (1991). Do doctors and patients agree? Views of the outcome of transurethral resection of the prostate. Int J Technol Assess Helath Care.

[8] Fränneby U, Gunnarsson U, Wollert S, G S (2005). Discordance between the patient’s and surgeon’s perception of complications following hernia surgery. Hernia.

[9] Haapaniemi S, Nilsson E (2002). Recurrence and pain three years after groin hernia repair. Validation of postal questionnaire and selective physical examination as a method of follow up. Eur J Surg.

[10] Iyer R, Gentry-Maharaj A, Nordin A, Liston R, Burnell M, Das N (2013). Patient-reporting improves estimates of postoperative complication rates: a prospective cohort study in gynaecological oncology. Br J Cancer.

[11] Mannion A, Fekete T, O’Riordan D, Porchet F, Mutter U, Jeszenszky D (2013). The assessment of complications after spine surgery: time for a paradigm shift?. Spine J.

[12] Grosse Frie K, van der Meulen J, Black N (2012). Relationship between patients’ reports of complications and symptoms, disability and quality of life after surgery. Br J Surg.

[13] Loui A, Robinson L, Bogue M, Hyde S, Forman S, Bhatia S (2000). Validation of self-reported complications by bone marrow transplantation survivors. Bone Marrow Transplant.

[14] Visser A, Ubbink D, Gouma D, Goslings J (2004). Surgeons are overlooking post-discharge complications: a prospective cohort study. World J Surg.

[15] Whitby M, McLaws M, Doidge S, Collopy B (2007). Post-discharge surgical site surveillance: does patient education improve reliability of diagnosis?. J Hosp Infect.

[16] Whitby M, McLaws ML, Collopy B, Looke D, Doidge S, Henderson B (2002). Post-discharge surveillance: can patients reliably diagnose surgical wound infections?. J Hosp Infect.

[17] Dushey C, Bornstein L, Alexiades M, Westrich G (2011). Short-term coagulation complications following total knee arthroplasty: a comparison of patient-reported and surgeon-verified complication rates. J Arthroplasty.

[18] Alazzawi S, Bardakos N, Hadfield S, Butt U, Beer Z, Field R (2012). Patient-reported complications after elective joint replacement surgery: are they correct?. J Bone Joint Surg Br.

[19] Greenbaum J, Bornstein L, Lyman S, Alexiades M, Westrich G (2012). The validity of self-report as a technique for measuring short-term complications after total hip arthroplasty in a joint replacement registry. J Arthroplasty.

[20] Rotondi M. kappaSize: Sample Size Estimation Functions for Studies of Interobserver Agreement. 2013. R package version 1.1. Available from: https://CRAN.R-project.org/package=kappaSize. Accessed 16 Sept 2016.

[21] R Core Team (2017). R: A language and environment for statistical computing.

[22] Heo SM. Reliability of patient reported complications: Can patients accurately report complications following hip or knee arthroplasty procedures? (monograph). 2016. 10.1186/s12874-018-0645-0 PMC6330452 30634917

[23] Desai A, Dramis A, Board T (2013). Leg length discrepancy after total hip arthroplasty: a review of literature. Curr Rev Musculoskelet Med.

[24] Zellmer C, Zimdars P, Parker S, Safdar N (2015). Leg length discrepancy after total hip arthroplasty: a review of literature. Am J Infect Control.

[25] Parimi N, Lane N, Bauer D, Hochberg M, Nevitt M (2010). Accuracy of self-reported diagnosis of hip replacement. Arthritis Care Res (Hoboken).

[26] AJ V, Garrett J (2005). Understanding interobserver agreement: the kappa statistic. Fam Med.

[27] McHugh M (2012). Interrater reliability: the kappa statistic. Biochem Med (Zagreb).

[28] Veen E, Steenbruggen J, Roukema J (2005). Classifying surgical complications: a critical appraisal. Arch Surg.

