# Supplementary material for: Reliability of patient-reported complications following hip or knee arthroplasty procedures
Source: BMC Med Res Methodol. 2019 Jan 11;19:15. doi: 10.1186/s12874-018-0645-0 (PMC6330452; doi:10.1186/s12874-018-0645-0)
Supplement: Supplementary file 1 — Full report on study with additional tables and R code. (PDF 732 kb) [file 12874_2018_645_MOESM1_ESM.pdf]

Reliability of patient reported complications: Can patients accurately report complications following hip or knee arthroplasty procedures?

Sung Mu Heo

University of New South Wales

Supervisor: A/Prof Justine Naylor

Co-Supervisors: Prof Ian Harris, Dr Timothy Churches

5405 words (including in-text citations)

Abstract

Introduction- Patient reported outcomes are increasingly used to assess the success of surgical procedures. Data regarding complications are often included as an outcome. However, these data must be validated to be accurate, and used in clinical practice.
Method- This was a retrospective descriptive study of 364 patients of six surgeons who had completed their six-month follow-up review questionnaire in the Arthroplasty Clinical Outcomes Registry, National (ACORN). Patient-reported complications (PRC) were compared to surgeon reported complications recorded in their medical files in their private consulting rooms. Validity and agreement scores were assessed.
Results- Patients returned overall low sensitivity (0.14), negative predictive value (NPV) (0.13) and kappa values (0.11), but very high specificity (0.98), positive predictive value (PPV) (0.98) and agreement values (96.31). Analyses performed categorising patients by surgeon, joint operated and time between surgery and follow-up review revealed insignificant differences between these groups.
Conclusion- Patients are accurate in reporting the absence of complications, but not the presence. Sensitivity of PRC needs to be improved. Greater attention to the clarity of the questions asked may help in this respect.

Acknowledgements

I would like to thank Professor Ian Harris for taking me on as a research student, his teachings regarding good research procedures and to always ask for evidence; Associate Professor Justine Naylor for guidance and support in the design and execution of my research; and Dr Timothy Churches for the countless hours spent going through and teaching me how to code, and reinforcing the importance of good research.

Besides my supervisors, I would like to thank the surgeons who agreed to participate in this study and my fellow research students at the Whitlam Orthopaedic Research Centre for a year of engaging discussions and all the fun that we have had.

Background

Total hip and knee arthroplasties (THA, TKA) have shown to be highly effective in relieving pain, restoring function and ensuring ongoing mobility in patients with advanced or end-stage arthritis of these joints (Bourne, Chesworth, Davis, Mahomed, & Charron, 2010). In Australia, the numbers of THA and TKA procedures being performed are increasing substantially, with 102,570 procedures, (44,710 hips, 57,860 knees) reported in the Australian Orthopaedic Association National Joint Replacement Registry in 2015 (AOANJRR, 2016). This represents an annual increase of 2.6% in hip procedures and 5.9% in knee procedures.

The success of these procedures has historically been evaluated on the basis of the survival of the prosthesis, implant revision rates and time between the initial surgery and implant removal (Arden et al., 2011; Ayers & Franklin, 2014). Although patient-centred (defined here as patient-reported) results are considered subjective, they are regarded as the most direct measurements of outcomes, and are of increasing relevance as patients' improvements in pain and function are recognised as appropriate success measures of surgery (WORC, 2016).

Many clinical outcome registries have been implemented to capture patient-reported outcome measures (PROMs) and are widely used to gauge treatment efficacy and assess the quality of these procedures (Lyman & Hidaka, 2016). Complication rates for THA and TKA procedures are relatively low, but are often included in registries as they are indicators of risks and quality of surgery, and are major factors in patients' assessment of surgery. These data can be analysed to help inform what is best practice to reduce the rates of these complications and associated costs (Capozzi & Rhodes, 2010; Lacny, Bohm, Hawker, Powell, & Marshall, 2016). Registries often contact patients directly, which is preferable to abstracting the required data from surgeon and general practitioner patient records as it is relatively inexpensive, time-saving, avoids intrusion of health professionals' schedules, whilst providing a window into understanding patients' perceived outcomes. However, to use patient-reported complications (PRC) to understand complication rates following surgery and influence current practice, the accuracy of the data must be assessed.

Current literature available regarding PRC validation have shown varying results, depending on surgery type and methodology. Studies involving patients undergoing general surgery reported lower sensitivity and PPVs but high specificity and NPVs and moderate agreement. Five other studies (two in hernia repair, one each in gynaecological oncology, prostatectomy and spinal surgery) all produced similar results, but with lower agreement (Black, 1991; Franneby, Gunnarsson, Wollert, & Sandblom, 2005; Haapaniemi, 2002; Iyer et

al., 2013; Mannion et al., 2013). A large observational study in England compared over 200,000 patients across four procedures (THA, TKA, inguinal hernia repair and varicose vein surgery) and confirmed the validity of PRC (Grosse Frie, van der Meulen, & Black, 2012). However, only 4 complications were assessed (allergy or reaction to drug, urinary problems, bleeding and wound problems), and complications specific to the different procedures were not investigated. One study involving bone marrow transplant patients found high sensitivity, specificity and agreement values when PRC were compared with surgeons, and on that basis recommended PRC as an appropriate replacement for surgeon reported complications (Loui, 2000).

There were four studies focussed on orthopaedic surgery patients, including the aforementioned large English study. Greenbaum *et al.* aimed to validate PRC in five complication types (pulmonary embolism, dislocation, fracture, deep vein thrombosis (DVT) and bleeding), and found that concordance varied between 32.0% and 88.9% depending on complication type (Greenbaum, Bornstein, Lyman, Alexiades, & Westrich, 2012). Lowest concordance was for major bleeding and highest was for pulmonary embolism. Alazzawi *et al.* conducted a similar study with more complication types, and reported similar results, ranging from 36.0% for numbness to 94.5% for infection (Alazzawi et al., 2012). Dushey *et al.* studied the validity of patient-reported coagulation complications, and saw that concordance was as low as 36.7% for major bleeding episodes and as high as 86.2% for DVT (Dushey, Bornstein, Alexiades, & Westrich, 2011). These studies have all suggested that the strength of concordance was low for complications as such as major bleeding and numbness as they are somewhat ambiguous events or outcomes which may be difficult for patients to identify definitively. Others, as such as fractures, venous-thromboembolic events, and infections were thought to produce higher concordance as they are major events, easily identified by patients, possibly due to the quite specific treatments required for them.

However, the orthopaedic studies collectively failed to assess patients who had not reported complications, rendering it impossible to calculate the classification performance or agreement statistics for these data. This means that although these studies can assess the accuracy of PRC for patients who reported complications, they cannot assess false negatives. Thus, a more complete study taking false negatives into account must be undertaken to better understand the validity of PRC.

Aim

This study aims to assess the reliability of PRC as a substitute for surgeon’s records through examining sensitivity, specificity and agreement values. We hypothesise that specificity will be higher than sensitivity when PRC are assessed against surgeon reported complications, and agreement values will be lower in subjective complications that rely on the patients’ opinions (e.g. pain) than more observable, objective complications (e.g. joint dislocation).

Methodology

This was a retrospective descriptive study within a larger longitudinal observational cohort study in the form of a registry across six hospitals in New South Wales.

Participants

Six surgeons with the highest volumes of TKA and THA procedures captured by the Arthroplasty Clinical Outcomes Registry, National (ACORN) were approached for this study, of whom all consented to participate. ACORN collects preoperative data from patients undergoing elective TKA or THA (inclusion criteria mentioned below) from eight institutions around New South Wales. This is done by site co-ordinators at each hospital, and the patient data are forwarded to the registry to be followed up six-months post-operatively. Follow-up questionnaires are administered by an interviewer over the phone. ACORN measures a range of outcomes, broadly grouped into general health, joint pain and function, patient-rated satisfaction and complications (WORC, 2016).

Ethical and consent concerns

All patients participating in ACORN gave informed written consent either prior to surgery, or immediately following, for their data to be included in a registry and for post-operative follow-up of their complications and outcomes via a questionnaire over telephone. The study reported here was approved for ethics by the Hunter New England Human Research Ethics Committee as an incorporated sub-study to improve services and outcomes for joint replacements for ACORN on 21/04/2016.

Sample size calculation

The appropriate sample size was estimated using the kappaSize package for the R statistical computing environment (R Core Team, 2013).A sample size with statistical power to detect a Cohen's *kappa* agreement statistic in in the range 0.4 to 0.7 with a complications prevalence (per category of complication) of 10%, with a standard *alpha* parameter of 0.05 was calculated to be a total of 300 patients. Individual complication rates recorded by the ACORN registry range from about 1% to over 15%. Ten percent was chosen as an approximate mean complications prevalence. This was deemed to provide an adequate margin of error to ensure that the study was not under-powered. Approximately fifty patients who have completed their six-month telephone follow-up were randomly selected from amongst the arthroplasty cases performed in 2015 for each of the six surgeons, using a random number generator function in a R programme.

Inclusion criteria

Patients were included in this study if they had completed their six-month follow-up interview with ACORN. Patients were included in ACORN if the person was 18 years of age or over, the arthroplasty (primary or revision) of the hip or knee was elective, the surgery was undertaken at a hospital participating in the registry, and the person was not cognitively impaired or unable to understand the process for participation.

Data collection

Information on post-discharge complications for each patient, as recorded by their treating surgeon, was abstracted from the clinical notes or medical records maintained by each surgeon in their private consultation rooms. Post-operative follow-up of almost all arthroplasty patients, including those treated in public-sector hospitals occur in these private consulting rooms. Thus, no selection bias was introduced by this means of collecting

surgeon-recorded follow-up data. The items to be abstracted were the same as those collected from each patient at the six-month post-operative follow-up interview (explained in detail below in *Post-operative follow-up*). The specific data items were as follows:

- Re-admission to hospital;
- Primary reason for re-admission;
- Same or different hospital re-admitted to;
- Reason for admission;
- Re-operation on replaced joint;
- Reason for re-operation;
- Complications (specific categories or other);
- Fact and date of death.

The lists of the randomly selected patients for each surgeon were prepared by one of the co-supervisors of this project (TC). This was done to ensure that the investigator (the author) was blinded to the results of the six-month follow-up interview for these patients in the ACORN database. This was to avoid the possibility of data-abstractor bias in the data abstraction process. Following the completion of data abstraction from surgeons' rooms and recording results into a database, these records were locked and unable to be changed.

Post-operative follow-up

An exact copy of the complications questionnaire form used at the six-month follow-up interview was used by the investigator in the surgeons' private rooms. The full form includes several subjective questions regarding PROMs, satisfaction and self-perceived success which were not included as these are not recorded by surgeons.

The complications questionnaire consisted of:

- Six-month readmission- Patients were asked to state if they had been readmitted to any hospital since discharge from acute care for management of the index join: *Yes / No / Unknown or not stated*. If *Yes*, patients were asked to state;
    - Primary reason for readmission: *Deep vein thrombosis (DVT) / Pulmonary Embolism (PE) / Manipulation under anaesthetic (MUA) / Dislocation / Surgical site infection (SSI) / Wound dehiscence / Other, Unknown or unstated.*
        - If *Other*, patients were asked to specify.
    - Hospital that they were readmitted into: *Same public hospital as surgery / Different public hospital but same health district / Different public hospital in other health district / Same private hospital as surgery / Other private hospital / Unknown or not stated.*
    - If they were readmitted to any hospital for any other reason: *Yes / No / Unknown or unstated*
    - Reason for admission mentioned above: *Cardiac / Kidney or bladder / Cancer / Other / Unknown or not stated*
- Six-month reoperation- Patients were asked to state if they have had a reoperation on the replaced joint(s) since discharge: *Yes / No / Unknown or not stated*. If *Yes*, patients were asked to state;
    - Reason for reoperation: *SSI requiring surgery with no prosthesis removal / SSI requiring surgery with prosthesis removal / Dislocation / Joint stiffness /*

Periprosthetic fracture / Implant fracture / Bleeding / Pain / Other / Unknown or not stated.
        - If *Other*, patients were asked to specify.
- Complications- Patients were asked to state if they had any other problem or complication not requiring readmission: *Yes / No / Unknown or not stated.*
    - If *Yes*, patients were asked to state: *SSI requiring oral antibiotics / SSI requiring intravenous (IV) antibiotics / DVT index leg / DVT other leg / DVT both legs / PE / Dislocation / Joint stiffness / Bladder infection or retention / Fracture / Unexpected pain / Cardiac / Stroke / Leg length discrepancy / Joint or lower limb swelling / Paraesthesia or numbness / Cellulitis / Neuropathy / Muscle weakness / Respiratory infection / Other / Unknown or not stated.*
        - If *Other*, patients were asked to specify.
- Death- Interviewees were asked if the patient had died since discharge: *Yes / No / Unknown or not stated.*
    - If *Yes*, interviewees were asked to specify date of death (if known).

A copy of the questionnaire used appears as Appendix 1.

Readmission, reoperation and twenty-two separate complications were considered in this study. Twenty of these complications (excluding *other* and *unknown or not stated*) were arranged into the following groups based on similarity, in order to additionally assess validity and agreement within broader categories, as shown in table 1.

Table 1. Complications grouped by category used for analysis

Category	Included complications
Thromboembolic events	DVT index leg, DVT other leg, DVT both legs, Pulmonary embolism.
Infections of surgical site	SSI requiring oral antibiotics, SSI requiring IV antibiotics, cellulitis.
Problems involving the joint	Joint stiffness, Fracture, Leg length discrepancy, Joint or lower leg swelling, Dislocation.
Medical complications	Bladder infection or retention, Cardiac, Stroke, Neuropathy, Respiratory infection
Subjective complications	Unexpected pain, Paraesthesia or numbness, Muscle weakness.

Statistical analysis

The data were analysed by calculating the sensitivity, specificity, positive predictive values (PPV), negative predictive values (NPV), percentage agreement and unweighted Cohen's Kappa coefficient. To investigate whether additional factors influence these agreement metrics, analyses were also performed with patients categorised by attending surgeons, the joint operated on, and the time between surgery and follow-up review (which is not always at six months for surgeon follow-up, unlike the ACORN follow-up).

Data collected from surgeons' private rooms were collected and managed using the Research Electronic Data Capture (REDCap) research database tool hosted by UNSW Medicine IT services. REDCap is a secure, web-based application designed to support data capture for research studies, providing a) an intuitive interface for validated data entry; b) audit trails for tracking data manipulation and export procedures; c) record locking to prevent accidental or deliberate *post hoc* data manipulation; d) automated export procedures for seamless data downloads to common statistical packages; and e) procedures for importing data from external sources (Harris et al., 2009)

Statistical analyses were completed using the R statistical computing software environment for statistical computing (R Core Team, 2013). Several add-on packages for R were used to carry out specific calculations. Data frame manipulations were performed utilising the *dplyr* package (Wickham, 2016). Sensitivity, specificity, PPV and NPV were calculated utilising the *caret* package (Kuhn, 2016). Percentage agreement and Cohen's Kappa coefficients were calculated utilising the *irr* package (Gamer, 2012).

The Cohen's Kappa is a measurement of inter-rater reliability, which adjusts for chance agreement between raters, which is a major limitation of the use of simple percentage agreement values. The statistic is based on the chi-square distribution and is be calculated by the formula:

$$Kappa(\kappa) = \frac{\mathrm{P(a)} - P(e)}{1 - P(e)}$$

Where P(a) is the probability of agreement and P(e) is chance agreement. The kappa coefficient is based on the chi-square table. Therefore, in reference to the following table from Komagata (Komagata, 2002):

Raters		*Y*		
	Category	*A*	*B*	Σ
X	*A*	$P(AA)$	$P(AB)$	$P(A_X)$
	B	$P(BA)$	$P(BB)$	$P(B_X)$
	Σ	$P(A_Y)$	$P(B_Y)$	

Chance agreement is given by:

$$P(e) = \left(\frac{P(A_x) + P(A_y)}{2}\right)^2 + \left(\frac{P(B_x) + P(B_y)}{2}\right)^2$$

Cohen's Kappa is interpreted categorically; as values less than or equal to 0 is denotes no agreement; 0.01-0.20 as slight agreement; 0.21-0.40 as fair agreement; 0.41-0.60 as moderate agreement; 0.61-.80 as substantial agreement, and; 0.81-1.00 as almost perfect agreement (Landis, 1977).

The full R program code used for data preparation and analysis appear as Appendix 2.

Results

Demographics

From the sampling process, 364 patients were selected to be cross-examined, of whom 340 patients had at least one recorded review with a surgeon within six months of surgery. Overall, there were more females than males, and more TKA than THA. No significant between-patient differences in characteristics were observed between surgeons. The largest difference was in follow-up time, which was driven by individual surgeon's usual practice. Surgeon identities have been suppressed in this report for confidentiality reasons.

Surgeons A, B and E reviewed the bulk of their patients within 8 weeks, whereas surgeons C, D and F reviewed closer to the six-month mark.

Table 2 summarises the characteristics of selected patients and time between follow-up consultations, compared by surgeon.

Table 2. Summary of patient characteristics

Demographics	A	B	C	D	E	F	Overall
Number	63	73	51	53	48	52	340
Males	20	26	19	16	17	20	118
Females	43	47	32	37	31	32	222
Mean age	68	67	67	69	72	68	68
SD	8.1	12.8	9.6	9.1	8.1	10.4	10.1
Joint							
Hips	11	25	21	16	13	16	102
Knees	52	48	30	37	35	36	238
Follow-up time							
<6 weeks	15	1	0	0	7	0	23
6-8 weeks	36	38	9	6	17	15	121
3-5 months	4	7	3	14	6	6	40
6 months	4	20	38	28	12	27	129
>12 months	4	7	1	5	5	4	26

Complications

A total of 163 complications were reported by 77 patients. Table 3 shows the patients grouped by the number of complications they reported.

Table 3. Total number of complications reported by patient

Number of reported complications	Patients	Total complications
0	263	0
1	32	32
2	23	46
3	10	30
4	6	24
5	5	25
6	1	6
	Total	163

Analysis of results

The results of the complete analysis are summarized in table 4.

Table 4. Validity and Agreement values for PRC when compared with surgeon notes[1].

Variable	n	TT[2]	TF[3]	FT[4]	FF[5]	Sensitivity	Specificity	PPV	NPV	Agreement %	kappa
**Readmission**	**340**	**9**	**9**	**7**	**315**	**0.50**	**0.98**	**0.56**	**0.97**	**95.29**	**0.50**
**Reoperation**	**340**	**3**	**2**	**2**	**333**	**0.60**	**0.99**	**0.60**	**0.99**	**98.82**	**0.59**
**Thromboembolic event**	**1360**	**1**	**3**	**4**	**1352**	**0.25**	**1.00**	**0.20**	**1.00**	**99.49**	**0.22**
DVT Index Leg	340	1	2	3	334	0.33	0.99	0.25	0.99	98.53	0.28
DVT Other Leg	340	0	0	0	340	NA	1.00	NA	NA	100.00	1.00
DVT Both Legs	340	0	0	0	340	NA	1.00	NA	NA	100.00	1.00
Pulmonary Embolism	340	0	1	1	338	0.00	1.00	0.00	1.00	99.41	0.00
**Infections**	**1020**	**2**	**13**	**5**	**1000**	**0.13**	**1.00**	**0.29**	**0.99**	**98.24**	**0.17**
SSI requiring Oral AB	340	2	9	5	324	0.18	0.98	0.29	0.97	95.88	0.20
SSI requiring Intravenous AB	340	0	0	0	340	NA	1.00	NA	NA	100.00	1.00
Cellulitis	340	0	4	0	336	0.00	1.00	NA	0.99	98.82	0.00
**Joint Problems**	**1700**	**6**	**31**	**66**	**1597**	**0.16**	**0.96**	**0.08**	**0.98**	**94.29**	**0.08**
Dislocation	340	0	0	0	340	NA	1.00	NA	NA	100.00	1.00
Stiffness	340	0	6	28	306	0.00	0.92	0.00	0.98	90.00	-0.03
Fracture	340	0	0	1	339	NA	1.00	NA	NA	99.71	0.00
Leg Length Discrepancy	340	1	9	4	326	0.10	0.99	0.20	0.97	96.18	0.12
Joint or Lower Leg Swelling	340	5	16	33	286	0.24	0.90	0.13	0.95	85.59	0.10
**Medical Complications**	**1700**	**1**	**11**	**5**	**1683**	**0.08**	**1.00**	**0.17**	**0.99**	**99.06**	**0.11**
Respiratory Infection	340	0	0	0	340	NA	1.00	NA	NA	100.00	1.00
Cardiac	340	0	0	1	339	NA	1.00	NA	NA	99.71	0.00
Stroke	340	0	0	0	340	NA	1.00	NA	NA	100.00	1.00
Bladder Infection/Retention	340	1	2	1	336	0.33	1.00	0.50	0.99	99.12	0.40
Neuropathy	340	0	9	3	328	0.00	0.99	0.00	0.97	96.47	-0.01
**Subjective Complications**	**1020**	**11**	**67**	**55**	**887**	**0.14**	**0.94**	**0.17**	**0.93**	**88.04**	**0.09**
Unexpected Pain	340	10	58	16	256	0.15	0.94	0.38	0.82	78.24	0.11
Paresthesia/Numbness	340	1	7	28	304	0.12	0.92	0.03	0.98	89.71	0.02
Muscle Weakness	340	0	2	11	327	0.00	0.97	0.00	0.99	96.18	-0.01
**Other**	**340**	**0**	**9**	**7**	**324**	**0.00**	**0.98**	**0.00**	**0.97**	**95.29**	**-0.02**
**Unknown**	**340**	**0**	**0**	**0**	**340**	**NA**	**1.00**	**NA**	**NA**	**100.00**	**1.00**
**Overall Complications[6]**	**7480**	**21**	**134**	**142**	**7183**	**0.14**	**0.98**	**0.13**	**0.98**	**96.31**	**0.11**

[1]The denominator for the combined complications is the number of subjects (340) multiplied by the number of individual complications in the combined category, and is thus a multiple of the number of subjects
[2]TT= True positives (Surgeons reported, patients reported)
[3]TF= False negatives (Surgeons reported, patients did not report)
[4]FT= False positives (Surgeons did not report, patients reported)
[5]FF= True negatives (Surgeons did not report, patients did not report)
[6]Overall excludes readmission and reoperation

We also examined the results when patients were categorized by surgeon, joint and follow-up time which are summarized in table 5.

Table 5. Validity and agreement values for PRC when compared with surgeon notes, categorised by surgeon, joint and follow up time

Variable	n	T/T	T/F	F/T	F/F	Sensitivity	Specificity	PPV	NPV	Agreement %	kappa
**Surgeon**											
Surgeon A	1386	3	12	39	1332	0.20	0.97	0.07	0.99	96.32	0.09
Surgeon B	1606	6	16	32	1552	0.27	0.98	0.16	0.99	97.01	0.19
Surgeon C	1122	3	16	17	1086	0.16	0.98	0.15	0.99	97.06	0.14
Surgeon D	1166	4	52	11	1099	0.07	0.99	0.27	0.95	94.60	0.09
Surgeon E	1056	1	13	26	1016	0.07	0.98	0.04	0.99	96.31	0.03
Surgeon F	1144	4	25	17	1098	0.14	0.98	0.19	0.98	96.33	0.14
**Joint**											
Hip	2244	5	46	16	2177	0.10	0.99	0.24	0.98	97.24	0.13
Knee	5236	16	88	126	5006	0.15	0.98	0.11	0.98	95.91	0.11
**Follow up time**											
<6 weeks	506	1	1	15	489	0.50	0.97	0.06	0.99	96.84	0.10
6-8 weeks	2662	3	19	44	2596	0.14	0.98	0.06	0.99	97.63	0.08
3-5 months	880	2	18	21	839	0.01	0.98	0.09	0.98	95.57	0.07
6 months	2838	13	82	50	2693	0.14	0.98	0.21	0.97	95.35	0.14
6-12 months	572	2	14	12	544	0.12	0.98	0.14	0.97	95.45	0.11

Validity values

Proportion of positive agreements (true/true, denoted TT) were low across all complications, with the highest rates observed for readmission (9) and unexpected pain (10). Highest rates of FT (surgeon/patient) disagreement were in stiffness (28), lower leg swelling (33) and paresthesia (28), whilst highest rates of TF (surgeon/patient) disagreement were in unexpected pain (58). There were high negative agreements (FF) rates throughout all complications due to the low prevalence of most complications. As a result, low values for sensitivities and PPV's and high values for specificities and NPV's were observed.

With the exception of readmission and reoperation, patients' sensitivity did not exceed 0.33 when compared to surgeons. Overall sensitivity was reported at 0.14, with nine complications having no result due to the lack of true positives and false negatives, and six

complications having zero sensitivity due to a lack of true positives. PPV were similar, with highest reported value of 0.38 and 0.13 overall.

In contrast, the lowest specificity value reported was 0.92, and 0.98 overall. NPV was equally high at 0.98 overall and all values were greater than 0.90, with the exception of unexpected pain (0.82). These results indicate that although patients accurately report that they did not have a complication, their ability to recall complications when they have in fact experienced one (according to their surgeon's records) is not very good.

Agreement values

High values of percentage agreement were observed in majority of the complications. Overall, the analysis revealed an agreement of 96%, with six complications that showed 100% agreement, fourteen complications between 95% and 100%, one complication between 90% and 95%, and three complications less than 90%. Categorically, subjective complications showed lowest agreement with 88%. Swelling, stiffness, unexpected pain and paraesthesia showed the lowest levels of agreement. Patients and surgeons unanimously reported no complications for the seven separate complications that were reported with 100% agreement.

Apart from the seven complications that had complete observed agreement (1.00), *kappa* values were low. Two complications were in fair agreement, five in slight agreement and eight showed no agreement. Values were as low as -0.03 (stiffness), and apart from bladder infection (0.40), values were lower than or equal to 0.28. Overall *kappa* value was 0.11.

By category

Analyses were also done by grouping patients by their surgeon, the operated joint and the time between surgery and follow-up review, in order to see if these variables were related to patients' ability to report complications accurately. Patients of surgeon B showed slightly higher sensitivity and kappa values, whilst patients of surgeon D showed slightly higher PPV values. Patients who had undergone TKA reported higher sensitivity whilst THA patients were more specific, and there were very slight differences when patients were categorised by follow-up period. Overall, no significant differences between these groupings of patients were observed.

Readmission and Reoperation

Readmission and reoperation showed higher sensitivity, PPV and kappa values than other complications. This was not a surprising finding, as they are major events that can be easily remembered by patients, and surgeons are provided with documentation from hospitals and referring doctors. The disparities, where they existed, may be due to patients being unable to differentiate between events related to the index joint and events due to comorbidities or unrelated conditions, that is, patients who were admitted to hospital for events unrelated to the arthroplasty procedure may have reported these as arthroplasty-related readmissions.

Patient deaths

No patients were reported as deceased at the time of data collection.

Discussion

Orthopaedic clinical registries are becoming increasingly popular due to their ability to monitor results of surgery in a time- and cost- efficient manner, whilst incorporating the patient's perspective in the assessment of their surgery. To use this information to influence current practice, however, the accuracy of this data must be assessed.

This study has demonstrated that when PRC data from a clinical registry is assessed against surgeon notes, they show high specificity, NPV and percentage agreement. These indicate that patients are able to accurately report that they did not experience any complications, as seen in the high true negative results. Since the rates of complications following THA and TKA procedures are extremely low, this study suggests that registries are adequately valid and reliable for assessing complication rates following TKA and THA procedures.

However, the very low sensitivity and PPV which was demonstrated in this study is in concordance with most of the evidence in the literature. Three studies on patients identifying surgical site infections and one on hernia repair patients showed that PRC typically showed lower PPV and sensitivity than NPV and specificity (Haapaniemi, 2002; Whitby et al., 2002; Whitby, McLaws, Doidge, & Collopy, 2007). Registries could be a much better tool for assessing complications if these values could be improved. This is difficult due to the low rates of complications, as small degrees of disagreement can have large effects on calculated sensitivity values and NPV, which in turn can be masked in the specificity and percentage agreement values due to the large number of true negative values (kappa coefficient is addressed in a later section). Nevertheless, if the true negative results were ignored, there were only 21 (7.07%) accounts of patients and surgeons agreeing on the presence and type of complications, in comparison to 276 (92.9%) accounts of disagreement, out of a total of 297 comparisons.

Where there were disagreeing reports, patients were more likely to over-report complications than surgeons, in most categories. High rates of inaccurate (or at least, discordant) reporting by patients for stiffness, paraesthesia and muscle weakness were expected, and consistent with the hypothesis that patients are more inconvenienced by minor complications than surgeons often believe. Patients were more likely to under-report leg length discrepancies and infections. This may be because patients are seldom bothered by minor differences in leg length and often overlook these as long as they have improved function, whereas surgeons regard unequal leg lengths as a sign of poor technique. Furthermore, the mean patient age for this study was 68 years, with most of the patients in an age range which is likely to be prescribed to multiple medications. To these patients, antibiotics may become "just another pill" and may possibly account for their under-reporting of low-level wound infection.

Currently, data for the ACORN is retrieved via a phone call questionnaire, and complications are enquired simply by a "yes" or a "no". This may be appropriate for complications as such as dislocation or infection, as any amount of either would constitute an adverse complication. However, in the case of complications as such as swelling, stiffness,

weakness, pain and paraesthesia, there is a degree of normality in these events following surgery as part of a natural healing process. These complications should not be assessed by merely presence or absence, but the time frame in which it happened and the degree of debility caused by them should be sought.

Dushey *et al.* noted similar deficiencies in questionnaires and proposed that quantitative or degree of seriousness criteria should be added when enquiring after the less objective complications (Dushey et al., 2011). A similar study on general surgery procedures interviewed patients by asking if "an adverse outcome had occurred between discharge and 30 days after discharge," and found that patients grossly over-reported complications as they would describe their symptoms (e.g. pain and fever) compared to surgeons who observed diagnoses (e.g. infection) (Visser, Ubbink, Gouma, & Goslings, 2014). Another study allowed patients to freely describe whether "any complications [arose] as a consequence of your operation three months ago?" They critiqued their own methodology, and discussed whether clear definitions would improve concordance rates (Mannion et al., 2013).

This study has found similar results and suggests that clarity in what defines a complication may decrease the high levels of disagreement which were observed. For example, neuropathy and numbness are very similar complications, but neuropathy was reported by more surgeons and patients were more likely to report numbness, perhaps forgivably so. If the nuanced differences between complications were explained more comprehensively to patients, they may be able to more accurately report these complications. Further, muscle weakness encompasses anything from slight difficulty in movement to complete immobilisation. If patients were presented with strict criteria for weakness that constituted a complication, for example, a score lesser than or equal to three in strength grading (sufficient muscle contraction to move joint through full range of motion against gravity, but not resistance), it may render higher values for PRC validity.

A surprising finding from this study was the high rates of false negative results for unexpected pain. Joint pain is a major reason for patients undergoing THA and TKA, and hence, it was expected that patients would over-report pain if it continued following their procedures. This may be an incorrect assumption, and the observed results may actually be because the questionnaire refers to this specifically as "unexpected pain", whilst surgeons (who do not follow a *pro forma* set of questions) may have noted any pain that the patient reported. This suggests that patients may in fact expect a certain amount of pain following surgery, and added measures of clarifying and quantifying these complications in questionnaires may help improve the accuracy of PRC. However, a study compared the ability of patients to accurately report surgical wound infections between a group of patients who were trained in local and systemic signs and symptoms of infection to a group who were not (Whitby et al., 2007). Interestingly, both groups reported identically high sensitivity (83.3), high specificity (98.1, 93.7) and NPV (97.6, 98.1), but the untrained group surprisingly showed higher PPV (83.3, 65.2). This study may indicate that patient education may not necessarily be a solution to improve patients' abilities to accurately report complications. However, it should be noted that this is a single study focussing on one type of complication, and the breadth of knowledge in this area is not great, thus further investigations are required to be able to make firm conclusions in this respect.

The three orthopaedic studies seeking to validate PRC were limited as they only assessed the accuracy of patients who reported complications, and not the accuracy of those who reported no complications. This both prevented them from measuring validity values as

such as sensitivity and specificity, and were prone to selection bias by only investigating the group of patients who reported complications. Two of these studies (Dushey et al., 2011; Greenbaum et al., 2012) stated referenced the same study by Parimi *et al.* (Parimi, Lane, Bauer, Hochberg, & Nevitt, 2010) which reported false negative rates of 0.28% in patients reporting simply if they had had a THA, and hypothesised that it would be similar with PRC. A strength of this study was that it investigated both groups, and was able to assess the ability of patients to accurately report when they did have a complication, as well as when they did not, and the findings of this study support the assumptions made by Dushey *et al.* and Greenbaum *et al.* as false negative rates were 1.9% (142/7480).

Although it is the most commonly reported measure of inter-observer agreement, the use of Cohen's kappa has been questioned in the literature (Viera, 2005). Jacob Cohen introduced the statistic to account for random or chance agreement, but the assumptions made about rater independence may overestimate chance agreement, thereby underestimating the agreement value (McHugh, 2012). This has been the finding in this study. Pulmonary embolism, for example, overall returned a percentage agreement of 99.41% but a kappa statistic of 0.00. However, there were 338 cases of agreement and only 2 cases of disagreement. In comparison, bladder infection and retention returned a kappa statistic of 0.40, when there was just one less case of agreement (337 agree: 3 disagree). This shows that the kappa statistic is not very consistent when both raters are reliable, and overcompensates for agreement by chance. Percentage agreement may therefore be a more appropriate measure when agreement by chance is negligible.

This study also sought to assess the effect of various variables in a patient's ability to report complications. However, despite minor differences, the effect of surgeon performing surgery, joint operated on and follow-up time between surgery and review were shown to be insignificant. Although the sensitivity value for patients reviewed at less than six weeks is higher at 0.50, this is slightly misleading as there was only one true positive and one false negative.

Limitations

There are several limitations to this study. Firstly, complications noted in surgeons' records were accepted as the gold standard for this study, which may have been an incorrect assumption. A Swiss study found that surgeons failed to report 71% of PRC, and 61% of complications reported by surgeons were not reported by patients (Iyer et al., 2013). Others have argued that patients are the most appropriate judge to evaluate post-operative treatment, as although surgeons may have a better idea about what were "true" medical and surgical complications, only the patients have the complete picture of the adverse events (Franneby et al., 2005; Mannion et al., 2013). Surgeons may also be prone to overlooking complications and keeping incomplete or inaccurate records (Visser et al., 2014).

Secondly, this study suffers to some degree of comparing "apples to oranges" as not all patients were followed up by surgeons at the six-month mark. This was addressed by grouping patients by follow-up period, so that patients reviewed six months' post-surgery could be isolated and compared. However, this showed no significant differences to other follow-up periods. A study where patients complete a questionnaire, then are immediately assessed by history and clinical examination by a surgeon would be ideal, as in Franneby's 2005 study (Franneby et al., 2005). However, the question asked in this study was whether PRC reported in registries are a reliable substitute for complications recorded in surgeons'

notes. This assumes, for example, that if a surgeon does not review a patient beyond six weeks post-operatively, they expect them to not experience any significant complications from that point onward. Hence, this raises the question of whether surgeons are an adequately complete source of information, as previously discussed, but does not challenge the validity of this study.

Lastly, it is possible that a small amount of bias may have been caused due to some cases being lost at follow up by both ACORN and surgeons. Twenty-four out of 364 (6.6%) patients were lost as there were no documented follow-up reviews by the surgeons, and in 2015, ACORN reported a loss of 2.6% of patients (WORC, 2016). Although these are small numbers, they may be significant in studies as such as this one, as patients who respond to these types of questionnaires may be more likely to report accurate complications. Louie et al. mentioned a similar limitation, as they based their study upon the first 100 of 212 respondents to their survey, noting that patients who promptly responded to the questionnaire may be more likely to accurately report complications, which may have led to results that were better than the true population (Loui, 2000). The study reported here used stratified random sampling, rather than sequential sampling as used by Loui *et al.*, and is thus superior in that respect. Furthermore, 8.0% of THA patients and 14.3% of TKA patients observed in ACORN reported low English proficiency in their questionnaire (WORC, 2016). Questionnaires in different languages that are more accessible to non-English speakers may help improve not only compliance rates, but also the accuracy of PRC. This is especially applicable in areas similar to the southern and south-western suburbs of Sydney, where there are people from many cultures and ethnicities in a single health district.

Conclusion

Accurate but efficient ascertainment of complication rates following surgery remains a highly important aspect of not only surgeon appraisal, but also of patient satisfaction and continuing improvements in medical care. The high concordance for true negative results along with high specificity, NPV and percentage agreement found in this study are encouraging, as it indicates that complication rates following THA and TKA are low, and PRC are accurate in this regard. However, the low sensitivity and PPV must be improved, and we suggest that improved wording and clarity of questionnaires used to retrieve data from patients in registries would aid in achieving this.

References

Alazzawi, S., Bardakos, N. V., Hadfield, S. G., Butt, U., Beer, Z. H., & Field, R. E. (2012). Patient-reported complications after elective joint replacement surgery: are they correct? *J Bone Joint Surg Br, 94*(8), 1120-1125. doi:10.1302/0301-620x.94b8.29040

AOANJRR. (2016). *Hip, Knee & Shoulder Arthroplasty Annual Report 2016*. Adelaide, Australia.

Arden, N. K., Kiran, A., Judge, A., Biant, L. C., Javaid, M. K., Murray, D. W., . . . Field, R. E. (2011). What is a good patient reported outcome after total hip replacement? *Osteoarthritis Cartilage, 19*(2), 155-162. doi:10.1016/j.joca.2010.10.004

Ayers, D. C., & Franklin, P. D. (2014). Joint replacement registries in the United States: a new paradigm. *J Bone Joint Surg Am, 96*(18), 1567-1569. doi:10.2106/JBJS.N.00641

Black, N., et al. (1991). Do doctors and patients agree? Views of the outcome of transurethral resection of the prostate. *Int J Technol Assess Helath Care, 7*(4), 533-544.

Bourne, R. B., Chesworth, B., Davis, A., Mahomed, N., & Charron, K. (2010). Comparing patient outcomes after THA and TKA: is there a difference? *Clin Orthop Relat Res, 468*(2), 542-546. doi:10.1007/s11999-009-1046-9

Capozzi, J. D., & Rhodes, R. (2010). Examining the ethical implications of an orthopaedic joint registry. *J Bone Joint Surg Am, 92*(5), 1330-1333. doi:10.2106/jbjs.i.01410

Dushey, C. H., Bornstein, L. J., Alexiades, M. M., & Westrich, G. H. (2011). Short-term coagulation complications following total knee arthroplasty: a comparison of patient-reported and surgeon-verified complication rates. *J Arthroplasty, 26*(8), 1338-1342. doi:10.1016/j.arth.2010.11.007

Franneby, U., Gunnarsson, U., Wollert, S., & Sandblom, G. (2005). Discordance between the patient's and surgeon's perception of complications following hernia surgery. *Hernia, 9*(2), 145-149. doi:10.1007/s10029-004-0310-x

Gamer, M., Lemon, J., Fellows, I., Singh, P. (2012). Package 'irr': Various Coefficients of INterrater Reliability and Agreement. *0.84*.

Greenbaum, J. N., Bornstein, L. J., Lyman, S., Alexiades, M. M., & Westrich, G. H. (2012). The validity of self-report as a technique for measuring short-term complications after total hip arthroplasty in a joint replacement registry. *J Arthroplasty, 27*(7), 1310-1315. doi:10.1016/j.arth.2011.10.031

Grosse Frie, K., van der Meulen, J., & Black, N. (2012). Relationship between patients' reports of complications and symptoms, disability and quality of life after surgery. *Br J Surg, 99*(8), 1156-1163. doi:10.1002/bjs.8830

Haapaniemi, S., Nilsson, E. (2002). Recurrence and pain three years after groin hernia repair. Validation of postal questionnaire and selective physical examination as a method of follow up. *Br J Surg, 168*(1), 22-28.

Harris, P. A., Taylor, R., Thielke, R., Payne, J., Gonzalez, N., & Conde, J. G. (2009). Research electronic data capture (REDCap)--a metadata-driven methodology and workflow process for providing translational research informatics support. *J Biomed Inform, 42*(2), 377-381. doi:10.1016/j.jbi.2008.08.010

Iyer, R., Gentry-Maharaj, A., Nordin, A., Liston, R., Burnell, M., Das, N., . . . Menon, U. (2013). Patient-reporting improves estimates of postoperative complication rates: a prospective cohort study in gynaecological oncology. *Br J Cancer, 109*(3), 623-632. doi:10.1038/bjc.2013.366

Komagata, N. (2002). Chance Agreement and Significance of the Kappa Statistic. Retrieved from http://nobo.komagata.net/pub/Komagata02-Kappa.pdf

Kuhn, M. e. a. (2016). Package 'caret': Classificaion and Regression Training. *6.0-71*.

Lacny, S., Bohm, E., Hawker, G., Powell, J., & Marshall, D. A. (2016). Assessing the comparability of hip arthroplasty registries in order to improve the recording and monitoring of outcome. *Bone Joint J, 98-b*(4), 442-451. doi:10.1302/0301-620x.98b4.36501

Landis, J. R., Koch, G.G. (1977). The Measurement of OBserver Agreement for Categorical Data. *Biometrics, 33*(1), 159-174. doi:10.2307/2529310

Loui, A. D., Robinson, L.L, Bogue, M., Hyde, S., Forman, S.J., Bhatia, S. (2000). Validation of self-reported complications by bone marrow transplantation survivors. *Bone Marrow Transplant, 25*(11), 1191-1196.

Lyman, S., & Hidaka, C. (2016). Patient-Reported Outcome Measures-What Data Do We Really Need? *J Arthroplasty, 31*(6), 1144-1147. doi:10.1016/j.arth.2016.01.073

Mannion, A. F., Fekete, T. F., O'Riordan, D., Porchet, F., Mutter, U. M., Jeszenszky, D., . . . Kleinstueck, F. S. (2013). The assessment of complications after spine surgery: time for a paradigm shift? *Spine J, 13*(6), 615-624. doi:10.1016/j.spinee.2013.01.047

McHugh, M. L. (2012). Interrater reliability: the kappa statistic. *Biochem Med (Zagreb), 22*(3), 276-282.

Parimi, N., Lane, N. E., Bauer, D., Hochberg, M. C., & Nevitt, M. C. (2010). Accuracy of self-reported diagnosis of hip replacement. *Arthritis Care Res (Hoboken), 62*(5), 719-724. doi:10.1002/acr.20111

R Core Team. (2013). *R: A language and environment for statistical computing* (http://www.r-project.org/ ed.). Vienna, Austria: R Foundation for Statistical Computing.

Viera, A. J. G., J.M. . (2005). Understanding interobserver agreement: the kappa statistic. *Family Medicine, 37*(5), 360-363.

Visser, A., Ubbink, D. T., Gouma, D. J., & Goslings, J. C. (2014). Surgeons are overlooking post-discharge complications: a prospective cohort study. *World J Surg, 38*(5), 1019-1025. doi:10.1007/s00268-013-2376-z

Whitby, M., McLaws, M. L., Collopy, B., Looke, D. F. L., Doidge, S., Henderson, B., . . . Sartor, A. (2002). Post-discharge surveillance: can patients reliably diagnose surgical wound infections? *Journal of Hospital Infection, 52*(3), 155-160. doi:10.1053/jhin.2002.1275

Whitby, M., McLaws, M. L., Doidge, S., & Collopy, B. (2007). Post-discharge surgical site surveillance: does patient education improve reliability of diagnosis? *J Hosp Infect, 66*(3), 237-242. doi:10.1016/j.jhin.2007.04.014

Wickham, H., Francois, R. (2016). Package 'dplyr': A Grammar of Data Manipulation. *0.5.0.*

WORC. (2016). ACORN Annual Report 2015. Retrieved from http://www.acornregistry.org/images/ACORN_AnnualReport_2015.pdf

Appendix

Appendix 1

ACORN 6-month follow-up data collection form;

Page 6-7: Readmission, Re-operation and Other Complications

WHITLAM
UNSW

Whitlam Orthopaedic Research Centre
Orthopaedic Department Locked Bag 7103 Liverpool BC NSW 1871
T: +61 2 8738 9254 F: +61 2 9602 7187 E: arthroplastyregistry@worc.org.au

Recovery from Surgery

Notes for Registry caller:

- Complications are required to have been treated
- Complications can be directly related to the index joint replacement procedure or another body system e.g. cardiac system

Six Month Readmission

Readmitted to any hospital since discharge from acute care for management of index joint ?
- ☐ Yes
- ☐ No
- ☐ Unknown/Not stated

Primary reason for readmission
- ☐ DVT
- ☐ PE
- ☐ MUA
- ☐ Dislocation
- ☐ SSI
- ☐ Wound dehiscence
- ☐ Other
- ☐ Unknown/Not stated

If other, specify: ____________________

Which hospital was the person readmitted to?
- ☐ Same public hospital as surgery
- ☐ Different public hospital same health district
- ☐ Different public hospital other health district
- ☐ Same private hospital as surgery
- ☐ Other private hospital
- ☐ Unknown/Not stated

Readmitted to any hospital for any other reason?
- ☐ Yes
- ☐ No
- ☐ Unknown/Not stated

Reason for admission
- ☐ Cardiac
- ☐ Kidney/bladder
- ☐ Cancer
- ☐ Other
- ☐ Unknown/Not stated

Six Month Reoperation

Reoperation on the replaced joint(s) since discharge?
- ☐ Yes
- ☐ No
- ☐ Unknown/Not stated

Reason for reoperation
- ☐ SSI requiring surgery with no prosthesis removal
- ☐ SSI requiring surgery with prosthesis removal
- ☐ Dislocation
- ☐ Joint stiffness
- ☐ Periprosthetic fracture
- ☐ Implant fracture
- ☐ Bleeding
- ☐ Pain
- ☐ Other
- ☐ Unknown/Not stated

If other, specify: ____________________

Page 6 of 7
Version 4, 1 September 2014
based on Version 3, 28 May 2013
Master_ACORN_6MonthFollow-up_ HIP_DataCollectionForm

Whitlam Orthopaedic Research Centre
Orthopaedic Department Locked Bag 7103 Liverpool BC NSW 1871
T: +61 2 8738 9254 F: +61 2 9602 7187 E: arthroplastyregistry@worc.org.au

Other Complications

Has the person experienced any of the following complications/problems not requiring readmission to hospital since discharge?

Has the person reported any other problem/complication not requiring readmission?	☐ Yes<br>☐ No<br>☐ Unknown/Not stated
If yes, specify from list<br><br>If other, specify:	☐ SSI requiring oral antibiotics<br>☐ SSI requiring IV antibiotics<br>☐ DVT index leg<br>☐ DVT other leg<br>☐ DVT both legs<br>☐ PE<br>☐ Dislocation<br>☐ Joint stiffness<br>☐ Bladder infection/retention<br>☐ Fracture<br>☐ Unexpected pain<br>☐ Cardiac<br>☐ Stroke<br>☐ Leg length discrepancy<br>☐ Joint or lower limb swelling<br>☐ Parasthesia/Numbness<br>☐ Cellulitis<br>☐ Neuropathy<br>☐ Muscle weakness<br>☐ Respiratory infection<br>☐ Other<br>☐ Unknown/Not stated
Has the patient died since discharge?	☐ Yes<br>☐ No<br>☐ Unknown/not stated
Date of death (if known):	______________________<br>(DD/MM/YYYY)

They are all the questions I have to ask you today.

Thank you very much for your time and assistance. Do you have any questions or queries regarding anything we might have discussed?

Comments:

(End conversation)

Appendix 2
R program code used in data preparation and analysis

```
# Read in data
vprc <- read.csv("REDCap_data/VPRC_DATA_LATEST.csv", stringsAsFactors = FALSE)
# use ACORN data loading code from Tim Churches
source("read-ACORN-Redcap-data.R")

# Loading packages required to library
library(data.table)
library(dplyr)
library(irr)
library(psych)
library(vcd)
library(knitr)
library(printr)
library(xtable)
library(caret)

# Join VPRC (surgeon) and ACORN (patient) data
vprc_joined <- left_join(vprc, ACORN, by="id_number")

# Checking left join worked.
nrow(vprc)
nrow(vprc_joined)

# Quick check that data corresponds after the join
# Columns ending in .x come from the VPRC data, those ending in .y come from ACORN
select(vprc_joined,pers_last_name.x, pers_last_name.y, pers_dob.x, pers_dob.y, pers_sex.x,
pers_sex.y)

identical(vprc_joined$pers_last_name.x, vprc_joined$pers_last_name.y)
identical(vprc_joined$pers_sex.x, vprc_joined$pers_sex.y)

# Corresponding columns formatted
vprc_joined$pers_sex.x <-
factor(vprc_joined$pers_sex.x,levels=c("1","2","999"),labels=c("Male","Female","Unknown/Not
stated"))

vprc_joined$readm_mth6_any_spec.x <- NA
many_to_one_col <- function(df, col, colval, newcol, newval) {
  col <- deparse(substitute(col))
  newcol <- deparse(substitute(newcol))
  if (length(df[col] == 1) > 0) {
    df[df[col] == colval,newcol] <- newval
  }
}

many_to_one_col(vprc_joined, readm_reason_other_reason___1, 1, readm_mth6_any_spec.x, "1")
many_to_one_col(vprc_joined, readm_reason_other_reason___2, 1, readm_mth6_any_spec.x, "2")
many_to_one_col(vprc_joined, readm_reason_other_reason___3, 1, readm_mth6_any_spec.x, "3")
many_to_one_col(vprc_joined, readm_reason_other_reason___4, 1, readm_mth6_any_spec.x, "89")
many_to_one_col(vprc_joined, readm_reason_other_reason___5, 1, readm_mth6_any_spec.x, "999")

vprc_joined$readm_mth6_any_spec.x =
factor(vprc_joined$readm_mth6_any_spec.x,levels=c("1","2","3","89","999"),labels=c("Cardiac","Kidney
/bladder","Cancer","Other","Unknown/Not stated"))
vprc_joined <- rename(vprc_joined, readm_mth6_any_spec.y = readm_mth6_any_spec)

vprc_joined <- rename(vprc_joined, reop_mth6.x = reop_post_discharge)
vprc_joined$reop_mth6.x = factor(vprc_joined$reop_mth6.x,levels=c(1,2),labels=c("Yes","No"))
vprc_joined <- rename(vprc_joined, reop_mth6.y = reop_mth6)
vprc_joined$reop_mth6.y =
factor(as.character(vprc_joined$reop_mth6.y),levels=c("Yes","No"),labels=c("Yes","No"))

vprc_joined$reop_mth6_reason.x <- NA
many_to_one_col(vprc_joined, reop_reason___1, 1, reop_mth6_reason.x, "1")
many_to_one_col(vprc_joined, reop_reason___2, 1, reop_mth6_reason.x, "2")
many_to_one_col(vprc_joined, reop_reason___3, 1, reop_mth6_reason.x, "3")
many_to_one_col(vprc_joined, reop_reason___4, 1, reop_mth6_reason.x, "4")
many_to_one_col(vprc_joined, reop_reason___5, 1, reop_mth6_reason.x, "5")
many_to_one_col(vprc_joined, reop_reason___6, 1, reop_mth6_reason.x, "6")
many_to_one_col(vprc_joined, reop_reason___7, 1, reop_mth6_reason.x, "7")
many_to_one_col(vprc_joined, reop_reason___8, 1, reop_mth6_reason.x, "8")
many_to_one_col(vprc_joined, reop_reason___9, 1, reop_mth6_reason.x, "999")

vprc_joined$joint.x = factor(vprc_joined$joint.x,levels=c(1,2),labels=c("Hip", "Knee"))

vprc_joined$flw_up_time = factor(vprc_joined$flw_up_time,levels=c(1,2,3,4,5,6),labels=c("Less than 6
weeks", "6 to 8 weeks", "3 to 5 months", "6 months", "7 to 12 months", "Greater than 12 months"))
```

```
vprc_joined$reop_mth6_reason.x =
factor(vprc_joined$reop_mth6_reason.x,levels=c("1","2","3","4","5","6","7","8","89","999"),labels=c(
"SSI requiring surgery with no prosthesis removal","SSI requiring surgery with prosthesis
removal","Dislocation","Joint stiffness","Periprosthetic fracture","Implant
fracture","Bleeding","Pain","Other","Unknown/Not stated"))
vprc_joined <- rename(vprc_joined, reop_mth6_reason.y = reop_mth6_reason)

identical(vprc_joined$pers_sex.x, vprc_joined$pers_sex.y)

vprc_joined$readm_mth6.x <- factor(vprc_joined$readmitted_6mths,levels=c(1,2),labels=c("Yes","No"))
vprc_joined <- rename(vprc_joined, readm_mth6.y = readm_mth6)
vprc_joined[vprc_joined$readm_mth6.y == "Unknown/Not stated",] <- NA
vprc_joined$readm_mth6.y <-
factor(vprc_joined$readm_mth6.y,levels=c("Yes","No"),labels=c("Yes","No"))

# Cleaning non-readmitted complications data
vprc_joined <- rename(vprc_joined, compl_mth6_ssi_oral_ab.x = comp_no_readm_spec___1)
vprc_joined$compl_mth6_ssi_oral_ab.x =
factor(vprc_joined$compl_mth6_ssi_oral_ab.x,levels=c(1,0),labels=c("True","False"), ordered=TRUE)
vprc_joined <- rename(vprc_joined, compl_mth6_ssi_oral_ab.y = compl_mth6_nonadm_spec___1)
vprc_joined$compl_mth6_ssi_oral_ab.y <-
factor(vprc_joined$compl_mth6_ssi_oral_ab.y,levels=c("True","False"),labels=c("True","False"),
ordered=TRUE)

vprc_joined <- rename(vprc_joined, compl_mth6_ssi_iv_ab.x = comp_no_readm_spec___2)
vprc_joined$compl_mth6_ssi_iv_ab.x =
factor(vprc_joined$compl_mth6_ssi_iv_ab.x,levels=c(1,0),labels=c("True","False"), ordered=TRUE)
vprc_joined <- rename(vprc_joined, compl_mth6_ssi_iv_ab.y = compl_mth6_nonadm_spec___2)
vprc_joined$compl_mth6_ssi_iv_ab.y <-
factor(vprc_joined$compl_mth6_ssi_iv_ab.y,levels=c("True","False"),labels=c("True","False"),
ordered=TRUE)

vprc_joined <- rename(vprc_joined, compl_mth6_dvt_index.x = comp_no_readm_spec___3)
vprc_joined$compl_mth6_dvt_index.x =
factor(vprc_joined$compl_mth6_dvt_index.x,levels=c(1,0),labels=c("True","False"), ordered=TRUE)
vprc_joined <- rename(vprc_joined, compl_mth6_dvt_index.y = compl_mth6_nonadm_spec___3)
vprc_joined$compl_mth6_dvt_index.y <-
factor(vprc_joined$compl_mth6_dvt_index.y,levels=c("True","False"),labels=c("True","False"),
ordered=TRUE)

vprc_joined <- rename(vprc_joined, compl_mth6_dvt_other.x = comp_no_readm_spec___4)
vprc_joined$compl_mth6_dvt_other.x =
factor(vprc_joined$compl_mth6_dvt_other.x,levels=c(1,0),labels=c("True","False"), ordered=TRUE)
vprc_joined <- rename(vprc_joined, compl_mth6_dvt_other.y = compl_mth6_nonadm_spec___4)
vprc_joined$compl_mth6_dvt_other.y <-
factor(vprc_joined$compl_mth6_dvt_other.y,levels=c("True","False"),labels=c("True","False"),
ordered=TRUE)

vprc_joined <- rename(vprc_joined, compl_mth6_dvt_both.x = comp_no_readm_spec___5)
vprc_joined$compl_mth6_dvt_both.x =
factor(vprc_joined$compl_mth6_dvt_both.x,levels=c(1,0),labels=c("True","False"), ordered=TRUE)
vprc_joined <- rename(vprc_joined, compl_mth6_dvt_both.y = compl_mth6_nonadm_spec___5)
vprc_joined$compl_mth6_dvt_both.y <-
factor(vprc_joined$compl_mth6_dvt_both.y,levels=c("True","False"),labels=c("True","False"),
ordered=TRUE)

vprc_joined <- rename(vprc_joined, compl_mth6_pe.x = comp_no_readm_spec___6)
vprc_joined$compl_mth6_pe.x =
factor(vprc_joined$compl_mth6_pe.x,levels=c(1,0),labels=c("True","False"), ordered=TRUE)
vprc_joined <- rename(vprc_joined, compl_mth6_pe.y = compl_mth6_nonadm_spec___6)
vprc_joined$compl_mth6_pe.y <-
factor(vprc_joined$compl_mth6_pe.y,levels=c("True","False"),labels=c("True","False"), ordered=TRUE)

vprc_joined <- rename(vprc_joined, compl_mth6_disl.x = comp_no_readm_spec___7)
vprc_joined$compl_mth6_disl.x =
factor(vprc_joined$compl_mth6_disl.x,levels=c(1,0),labels=c("True","False"), ordered=TRUE)
vprc_joined <- rename(vprc_joined, compl_mth6_disl.y = compl_mth6_nonadm_spec___7)
vprc_joined$compl_mth6_disl.y <-
factor(vprc_joined$compl_mth6_disl.y,levels=c("True","False"),labels=c("True","False"),
ordered=TRUE)

vprc_joined <- rename(vprc_joined, compl_mth6_stif.x = comp_no_readm_spec___8)
vprc_joined$compl_mth6_stif.x =
factor(vprc_joined$compl_mth6_stif.x,levels=c(1,0),labels=c("True","False"), ordered=TRUE)
vprc_joined <- rename(vprc_joined, compl_mth6_stif.y = compl_mth6_nonadm_spec___8)
vprc_joined$compl_mth6_stif.y <-
factor(vprc_joined$compl_mth6_stif.y,levels=c("True","False"),labels=c("True","False"),
ordered=TRUE)

vprc_joined <- rename(vprc_joined, compl_mth6_blad.x = comp_no_readm_spec___9)
```

```
vprc_joined$compl_mth6_blad.x =
factor(vprc_joined$compl_mth6_blad.x,levels=c(1,0),labels=c("True","False"), ordered=TRUE)
vprc_joined <- rename(vprc_joined, compl_mth6_blad.y = compl_mth6_nonadm_spec___9)
vprc_joined$compl_mth6_blad.y <-
factor(vprc_joined$compl_mth6_blad.y,levels=c("True","False"),labels=c("True","False"),
ordered=TRUE)

vprc_joined <- rename(vprc_joined, compl_mth6_frac.x = comp_no_readm_spec___10)
vprc_joined$compl_mth6_frac.x =
factor(vprc_joined$compl_mth6_frac.x,levels=c(1,0),labels=c("True","False"), ordered=TRUE)
vprc_joined <- rename(vprc_joined, compl_mth6_frac.y = compl_mth6_nonadm_spec___10)
vprc_joined$compl_mth6_frac.y <-
factor(vprc_joined$compl_mth6_frac.y,levels=c("True","False"),labels=c("True","False"),
ordered=TRUE)

vprc_joined <- rename(vprc_joined, compl_mth6_pain.x = comp_no_readm_spec___11)
vprc_joined$compl_mth6_pain.x =
factor(vprc_joined$compl_mth6_pain.x,levels=c(1,0),labels=c("True","False"), ordered=TRUE)
vprc_joined <- rename(vprc_joined, compl_mth6_pain.y = compl_mth6_nonadm_spec___11)
vprc_joined$compl_mth6_pain.y <-
factor(vprc_joined$compl_mth6_pain.y,levels=c("True","False"),labels=c("True","False"),
ordered=TRUE)

vprc_joined <- rename(vprc_joined, compl_mth6_card.x = comp_no_readm_spec___12)
vprc_joined$compl_mth6_card.x =
factor(vprc_joined$compl_mth6_card.x,levels=c(1,0),labels=c("True","False"), ordered=TRUE)
vprc_joined <- rename(vprc_joined, compl_mth6_card.y = compl_mth6_nonadm_spec___12)
vprc_joined$compl_mth6_card.y <-
factor(vprc_joined$compl_mth6_card.y,levels=c("True","False"),labels=c("True","False"),
ordered=TRUE)

vprc_joined <- rename(vprc_joined, compl_mth6_stro.x = comp_no_readm_spec___13)
vprc_joined$compl_mth6_stro.x =
factor(vprc_joined$compl_mth6_stro.x,levels=c(1,0),labels=c("True","False"), ordered=TRUE)
vprc_joined <- rename(vprc_joined, compl_mth6_stro.y = compl_mth6_nonadm_spec___13)
vprc_joined$compl_mth6_stro.y <-
factor(vprc_joined$compl_mth6_stro.y,levels=c("True","False"),labels=c("True","False"),
ordered=TRUE)

vprc_joined <- rename(vprc_joined, compl_mth6_legl.x = comp_no_readm_spec___14)
vprc_joined$compl_mth6_legl.x =
factor(vprc_joined$compl_mth6_legl.x,levels=c(1,0),labels=c("True","False"), ordered=TRUE)
vprc_joined <- rename(vprc_joined, compl_mth6_legl.y = compl_mth6_nonadm_spec___14)
vprc_joined$compl_mth6_legl.y <-
factor(vprc_joined$compl_mth6_legl.y,levels=c("True","False"),labels=c("True","False"),
ordered=TRUE)

vprc_joined <- rename(vprc_joined, compl_mth6_swel.x = comp_no_readm_spec___15)
vprc_joined$compl_mth6_swel.x =
factor(vprc_joined$compl_mth6_swel.x,levels=c(1,0),labels=c("True","False"), ordered=TRUE)
vprc_joined <- rename(vprc_joined, compl_mth6_swel.y = compl_mth6_nonadm_spec___15)
vprc_joined$compl_mth6_swel.y <-
factor(vprc_joined$compl_mth6_swel.y,levels=c("True","False"),labels=c("True","False"),
ordered=TRUE)

vprc_joined <- rename(vprc_joined, compl_mth6_numb.x = comp_no_readm_spec___16)
vprc_joined$compl_mth6_numb.x =
factor(vprc_joined$compl_mth6_numb.x,levels=c(1,0),labels=c("True","False"), ordered=TRUE)
vprc_joined <- rename(vprc_joined, compl_mth6_numb.y = compl_mth6_nonadm_spec___16)
vprc_joined$compl_mth6_numb.y <-
factor(vprc_joined$compl_mth6_numb.y,levels=c("True","False"),labels=c("True","False"),
ordered=TRUE)

vprc_joined <- rename(vprc_joined, compl_mth6_cell.x = comp_no_readm_spec___17)
vprc_joined$compl_mth6_cell.x =
factor(vprc_joined$compl_mth6_cell.x,levels=c(1,0),labels=c("True","False"), ordered=TRUE)
vprc_joined <- rename(vprc_joined, compl_mth6_cell.y = compl_mth6_nonadm_spec___17)
vprc_joined$compl_mth6_cell.y <-
factor(vprc_joined$compl_mth6_cell.y,levels=c("True","False"),labels=c("True","False"),
ordered=TRUE)

vprc_joined <- rename(vprc_joined, compl_mth6_neur.x = comp_no_readm_spec___18)
vprc_joined$compl_mth6_neur.x =
factor(vprc_joined$compl_mth6_neur.x,levels=c(1,0),labels=c("True","False"), ordered=TRUE)
vprc_joined <- rename(vprc_joined, compl_mth6_neur.y = compl_mth6_nonadm_spec___18)
vprc_joined$compl_mth6_neur.y <-
factor(vprc_joined$compl_mth6_neur.y,levels=c("True","False"),labels=c("True","False"),
ordered=TRUE)

vprc_joined <- rename(vprc_joined, compl_mth6_weak.x = comp_no_readm_spec___19)
vprc_joined$compl_mth6_weak.x =
factor(vprc_joined$compl_mth6_weak.x,levels=c(1,0),labels=c("True","False"), ordered=TRUE)
```

```
vprc_joined <- rename(vprc_joined, compl_mth6_weak.y = compl_mth6_nonadm_spec___19)
vprc_joined$compl_mth6_weak.y <-
factor(vprc_joined$compl_mth6_weak.y,levels=c("True","False"),labels=c("True","False"),
ordered=TRUE)

vprc_joined <- rename(vprc_joined, compl_mth6_resp.x = comp_no_readm_spec___20)
vprc_joined$compl_mth6_resp.x =
factor(vprc_joined$compl_mth6_resp.x,levels=c(1,0),labels=c("True","False"), ordered=TRUE)
vprc_joined <- rename(vprc_joined, compl_mth6_resp.y = compl_mth6_nonadm_spec___20)
vprc_joined$compl_mth6_resp.y <-
factor(vprc_joined$compl_mth6_resp.y,levels=c("True","False"),labels=c("True","False"),
ordered=TRUE)

vprc_joined <- rename(vprc_joined, compl_mth6_othe.x = comp_no_readm_spec___21)
vprc_joined$compl_mth6_othe.x =
factor(vprc_joined$compl_mth6_othe.x,levels=c(1,0),labels=c("True","False"), ordered=TRUE)
vprc_joined <- rename(vprc_joined, compl_mth6_othe.y = compl_mth6_nonadm_spec___89)
vprc_joined$compl_mth6_othe.y <-
factor(vprc_joined$compl_mth6_othe.y,levels=c("True","False"),labels=c("True","False"),
ordered=TRUE)

vprc_joined <- rename(vprc_joined, compl_mth6_unkn.x = comp_no_readm_spec___22)
vprc_joined$compl_mth6_unkn.x =
factor(vprc_joined$compl_mth6_unkn.x,levels=c(1,0),labels=c("True","False"), ordered=TRUE)
vprc_joined <- rename(vprc_joined, compl_mth6_unkn.y = compl_mth6_nonadm_spec___999)
vprc_joined$compl_mth6_unkn.y <-
factor(vprc_joined$compl_mth6_unkn.y,levels=c("True","False"),labels=c("True","False"),
ordered=TRUE)

vprc_followed_up <- vprc_joined[!is.na(vprc_joined$readm_mth6.x),]

# Grouped complications
## Thromboembolic
vprc_followed_up_thromboembolic <- vprc_followed_up
vprc_followed_up_thromboembolic$compl_mth6_thromboembolic.x <-
vprc_followed_up$compl_mth6_dvt_index.x
vprc_followed_up_thromboembolic$compl_mth6_thromboembolic.y <-
vprc_followed_up$compl_mth6_dvt_index.y

temp <- vprc_followed_up
temp$compl_mth6_thromboembolic.x <- vprc_followed_up$compl_mth6_dvt_other.x
temp$compl_mth6_thromboembolic.y <- vprc_followed_up$compl_mth6_dvt_other.y
vprc_followed_up_thromboembolic <- bind_rows(vprc_followed_up_thromboembolic, temp)

temp <- vprc_followed_up
temp$compl_mth6_thromboembolic.x <- vprc_followed_up$compl_mth6_dvt_both.x
temp$compl_mth6_thromboembolic.y <- vprc_followed_up$compl_mth6_dvt_both.y
vprc_followed_up_thromboembolic <- bind_rows(vprc_followed_up_thromboembolic, temp)

temp <- vprc_followed_up
temp$compl_mth6_thromboembolic.x <- vprc_followed_up$compl_mth6_pe.x
temp$compl_mth6_thromboembolic.y <- vprc_followed_up$compl_mth6_pe.y
vprc_followed_up_thromboembolic <- bind_rows(vprc_followed_up_thromboembolic, temp)

## Surgical site infection
vprc_followed_up_infection <- vprc_followed_up
vprc_followed_up_infection$compl_mth6_infection.x <- vprc_followed_up$compl_mth6_ssi_oral_ab.x
vprc_followed_up_infection$compl_mth6_infection.y <- vprc_followed_up$compl_mth6_ssi_oral_ab.y

temp <- vprc_followed_up
temp$compl_mth6_infection.x <- vprc_followed_up$compl_mth6_ssi_iv_ab.x
temp$compl_mth6_infection.y <- vprc_followed_up$compl_mth6_ssi_iv_ab.y
vprc_followed_up_infection <- bind_rows(vprc_followed_up_infection, temp)

temp <- vprc_followed_up
temp$compl_mth6_infection.x <- vprc_followed_up$compl_mth6_cell.x
temp$compl_mth6_infection.y <- vprc_followed_up$compl_mth6_cell.y
vprc_followed_up_infection <- bind_rows(vprc_followed_up_infection, temp)

## Joint problems
vprc_followed_up_joint <- vprc_followed_up
vprc_followed_up_joint$compl_mth6_joint.x <- vprc_followed_up$compl_mth6_disl.x
vprc_followed_up_joint$compl_mth6_joint.y <- vprc_followed_up$compl_mth6_disl.y

temp <- vprc_followed_up
temp$compl_mth6_joint.x <- vprc_followed_up$compl_mth6_stif.x
temp$compl_mth6_joint.y <- vprc_followed_up$compl_mth6_stif.y
vprc_followed_up_joint <- bind_rows(vprc_followed_up_joint, temp)

temp <- vprc_followed_up
temp$compl_mth6_joint.x <- vprc_followed_up$compl_mth6_frac.x
temp$compl_mth6_joint.y <- vprc_followed_up$compl_mth6_frac.y
```

```
vprc_followed_up_joint <- bind_rows(vprc_followed_up_joint, temp)

temp <- vprc_followed_up
temp$compl_mth6_joint.x <- vprc_followed_up$compl_mth6_legl.x
temp$compl_mth6_joint.y <- vprc_followed_up$compl_mth6_legl.y
vprc_followed_up_joint <- bind_rows(vprc_followed_up_joint, temp)

temp <- vprc_followed_up
temp$compl_mth6_joint.x <- vprc_followed_up$compl_mth6_swel.x
temp$compl_mth6_joint.y <- vprc_followed_up$compl_mth6_swel.y
vprc_followed_up_joint <- bind_rows(vprc_followed_up_joint, temp)

## Medical complications
vprc_followed_up_internal <- vprc_followed_up
vprc_followed_up_internal$compl_mth6_internal.x <- vprc_followed_up$compl_mth6_blad.x
vprc_followed_up_internal$compl_mth6_internal.y <- vprc_followed_up$compl_mth6_blad.y

temp <- vprc_followed_up
temp$compl_mth6_internal.x <- vprc_followed_up$compl_mth6_card.x
temp$compl_mth6_internal.y <- vprc_followed_up$compl_mth6_card.y
vprc_followed_up_internal <- bind_rows(vprc_followed_up_internal, temp)

temp <- vprc_followed_up
temp$compl_mth6_internal.x <- vprc_followed_up$compl_mth6_stro.x
temp$compl_mth6_internal.y <- vprc_followed_up$compl_mth6_stro.y
vprc_followed_up_internal <- bind_rows(vprc_followed_up_internal, temp)

temp <- vprc_followed_up
temp$compl_mth6_internal.x <- vprc_followed_up$compl_mth6_neur.x
temp$compl_mth6_internal.y <- vprc_followed_up$compl_mth6_neur.y
vprc_followed_up_internal <- bind_rows(vprc_followed_up_internal, temp)

temp <- vprc_followed_up
temp$compl_mth6_internal.x <- vprc_followed_up$compl_mth6_resp.x
temp$compl_mth6_internal.y <- vprc_followed_up$compl_mth6_resp.y
vprc_followed_up_internal <- bind_rows(vprc_followed_up_internal, temp)

## Subjective complications
vprc_followed_up_subjective <- vprc_followed_up
vprc_followed_up_subjective$compl_mth6_subjective.x <- vprc_followed_up$compl_mth6_pain.x
vprc_followed_up_subjective$compl_mth6_subjective.y <- vprc_followed_up$compl_mth6_pain.y

temp <- vprc_followed_up
temp$compl_mth6_subjective.x <- vprc_followed_up$compl_mth6_weak.x
temp$compl_mth6_subjective.y <- vprc_followed_up$compl_mth6_weak.y
vprc_followed_up_subjective <- bind_rows(vprc_followed_up_subjective, temp)

temp <- vprc_followed_up
temp$compl_mth6_subjective.x <- vprc_followed_up$compl_mth6_numb.x
temp$compl_mth6_subjective.y <- vprc_followed_up$compl_mth6_numb.y
vprc_followed_up_subjective <- bind_rows(vprc_followed_up_subjective, temp)

## Overall complications
vprc_followed_up_overall <- vprc_followed_up
vprc_followed_up_overall$compl_mth6_overall.x <- vprc_followed_up$compl_mth6_ssi_oral_ab.x
vprc_followed_up_overall$compl_mth6_overall.y <- vprc_followed_up$compl_mth6_ssi_oral_ab.y

temp <- vprc_followed_up
temp$compl_mth6_overall.x <- vprc_followed_up$compl_mth6_ssi_iv_ab.x
temp$compl_mth6_overall.y <- vprc_followed_up$compl_mth6_ssi_iv_ab.y
vprc_followed_up_overall <- bind_rows(vprc_followed_up_overall, temp)

temp <- vprc_followed_up
temp$compl_mth6_overall.x <- vprc_followed_up$compl_mth6_dvt_index.x
temp$compl_mth6_overall.y <- vprc_followed_up$compl_mth6_dvt_index.y
vprc_followed_up_overall <- bind_rows(vprc_followed_up_overall, temp)

temp <- vprc_followed_up
temp$compl_mth6_overall.x <- vprc_followed_up$compl_mth6_dvt_other.x
temp$compl_mth6_overall.y <- vprc_followed_up$compl_mth6_dvt_other.y
vprc_followed_up_overall <- bind_rows(vprc_followed_up_overall, temp)

temp <- vprc_followed_up
temp$compl_mth6_overall.x <- vprc_followed_up$compl_mth6_dvt_both.x
temp$compl_mth6_overall.y <- vprc_followed_up$compl_mth6_dvt_both.y
vprc_followed_up_overall <- bind_rows(vprc_followed_up_overall, temp)

temp <- vprc_followed_up
temp$compl_mth6_overall.x <- vprc_followed_up$compl_mth6_pe.x
temp$compl_mth6_overall.y <- vprc_followed_up$compl_mth6_pe.y
vprc_followed_up_overall <- bind_rows(vprc_followed_up_overall, temp)
```

```
temp <- vprc_followed_up
temp$compl_mth6_overall.x <- vprc_followed_up$compl_mth6_disl.x
temp$compl_mth6_overall.y <- vprc_followed_up$compl_mth6_disl.y
vprc_followed_up_overall <- bind_rows(vprc_followed_up_overall, temp)

temp <- vprc_followed_up
temp$compl_mth6_overall.x <- vprc_followed_up$compl_mth6_stif.x
temp$compl_mth6_overall.y <- vprc_followed_up$compl_mth6_stif.y
vprc_followed_up_overall <- bind_rows(vprc_followed_up_overall, temp)

temp <- vprc_followed_up
temp$compl_mth6_overall.x <- vprc_followed_up$compl_mth6_blad.x
temp$compl_mth6_overall.y <- vprc_followed_up$compl_mth6_blad.y
vprc_followed_up_overall <- bind_rows(vprc_followed_up_overall, temp)

temp <- vprc_followed_up
temp$compl_mth6_overall.x <- vprc_followed_up$compl_mth6_frac.x
temp$compl_mth6_overall.y <- vprc_followed_up$compl_mth6_frac.y
vprc_followed_up_overall <- bind_rows(vprc_followed_up_overall, temp)

temp <- vprc_followed_up
temp$compl_mth6_overall.x <- vprc_followed_up$compl_mth6_pain.x
temp$compl_mth6_overall.y <- vprc_followed_up$compl_mth6_pain.y
vprc_followed_up_overall <- bind_rows(vprc_followed_up_overall, temp)

temp <- vprc_followed_up
temp$compl_mth6_overall.x <- vprc_followed_up$compl_mth6_card.x
temp$compl_mth6_overall.y <- vprc_followed_up$compl_mth6_card.y
vprc_followed_up_overall <- bind_rows(vprc_followed_up_overall, temp)

temp <- vprc_followed_up
temp$compl_mth6_overall.x <- vprc_followed_up$compl_mth6_stro.x
temp$compl_mth6_overall.y <- vprc_followed_up$compl_mth6_stro.y
vprc_followed_up_overall <- bind_rows(vprc_followed_up_overall, temp)

temp <- vprc_followed_up
temp$compl_mth6_overall.x <- vprc_followed_up$compl_mth6_legl.x
temp$compl_mth6_overall.y <- vprc_followed_up$compl_mth6_legl.y
vprc_followed_up_overall <- bind_rows(vprc_followed_up_overall, temp)

temp <- vprc_followed_up
temp$compl_mth6_overall.x <- vprc_followed_up$compl_mth6_swel.x
temp$compl_mth6_overall.y <- vprc_followed_up$compl_mth6_swel.y
vprc_followed_up_overall <- bind_rows(vprc_followed_up_overall, temp)

temp <- vprc_followed_up
temp$compl_mth6_overall.x <- vprc_followed_up$compl_mth6_numb.x
temp$compl_mth6_overall.y <- vprc_followed_up$compl_mth6_numb.y
vprc_followed_up_overall <- bind_rows(vprc_followed_up_overall, temp)

temp <- vprc_followed_up
temp$compl_mth6_overall.x <- vprc_followed_up$compl_mth6_cell.x
temp$compl_mth6_overall.y <- vprc_followed_up$compl_mth6_cell.y
vprc_followed_up_overall <- bind_rows(vprc_followed_up_overall, temp)

temp <- vprc_followed_up
temp$compl_mth6_overall.x <- vprc_followed_up$compl_mth6_neur.x
temp$compl_mth6_overall.y <- vprc_followed_up$compl_mth6_neur.y
vprc_followed_up_overall <- bind_rows(vprc_followed_up_overall, temp)

temp <- vprc_followed_up
temp$compl_mth6_overall.x <- vprc_followed_up$compl_mth6_weak.x
temp$compl_mth6_overall.y <- vprc_followed_up$compl_mth6_weak.y
vprc_followed_up_overall <- bind_rows(vprc_followed_up_overall, temp)

temp <- vprc_followed_up
temp$compl_mth6_overall.x <- vprc_followed_up$compl_mth6_resp.x
temp$compl_mth6_overall.y <- vprc_followed_up$compl_mth6_resp.y
vprc_followed_up_overall <- bind_rows(vprc_followed_up_overall, temp)

temp <- vprc_followed_up
temp$compl_mth6_overall.x <- vprc_followed_up$compl_mth6_othe.x
temp$compl_mth6_overall.y <- vprc_followed_up$compl_mth6_othe.y
vprc_followed_up_overall <- bind_rows(vprc_followed_up_overall, temp)

temp <- vprc_followed_up
temp$compl_mth6_overall.x <- vprc_followed_up$compl_mth6_unkn.x
temp$compl_mth6_overall.y <- vprc_followed_up$compl_mth6_unkn.y
vprc_followed_up_overall <- bind_rows(vprc_followed_up_overall, temp)

a <- vprc_followed_up %>% group_by(id_number) %>%
summarise(num_of_complications.y=sum(compl_mth6_ssi_oral_ab.y == "True",
```

```
                                                # readm_mth6.y == "True" ,
                                                # reop_mth6.y == "True" ,
                                                compl_mth6_ssi_oral_ab.y == "True" ,
                                                compl_mth6_ssi_iv_ab.y == "True" ,
                                                compl_mth6_dvt_index.y == "True" ,
                                                compl_mth6_dvt_other.y == "True" ,
                                                compl_mth6_dvt_both.y == "True" ,
                                                compl_mth6_pe.y == "True" ,
                                                compl_mth6_disl.y == "True" ,
                                                compl_mth6_stif.y == "True" ,
                                                compl_mth6_blad.y == "True" ,
                                                compl_mth6_frac.y == "True" ,
                                                compl_mth6_pain.y == "True" ,
                                                compl_mth6_card.y == "True" ,
                                                compl_mth6_stro.y == "True" ,
                                                compl_mth6_legl.y == "True" ,
                                                compl_mth6_swel.y == "True" ,
                                                compl_mth6_numb.y == "True" ,
                                                compl_mth6_cell.y == "True" ,
                                                compl_mth6_neur.y == "True" ,
                                                compl_mth6_weak.y == "True" ,
                                                compl_mth6_resp.y == "True" ,
                                                compl_mth6_othe.y == "True" ,
                                                compl_mth6_unkn.y == "True" ,na.rm=TRUE))

table(a$num_of_complications.y,exclude=NULL)

vprc_followed_up$num_of_complications.y <- with(vprc_followed_up,sum(
                                                # readm_mth6.y == "True" ,
                                                # reop_mth6.y == "True" ,
                                                compl_mth6_ssi_oral_ab.y == "True",
                                                compl_mth6_ssi_iv_ab.y == "True" ,
                                                compl_mth6_dvt_index.y == "True" ,
                                                compl_mth6_dvt_other.y == "True" ,
                                                compl_mth6_dvt_both.y == "True" ,
                                                compl_mth6_pe.y == "True" ,
                                                compl_mth6_disl.y == "True" ,
                                                compl_mth6_stif.y == "True" ,
                                                compl_mth6_blad.y == "True" ,
                                                compl_mth6_frac.y == "True" ,
                                                compl_mth6_pain.y == "True" ,
                                                compl_mth6_card.y == "True" ,
                                                compl_mth6_stro.y == "True" ,
                                                compl_mth6_legl.y == "True" ,
                                                compl_mth6_swel.y == "True" ,
                                                compl_mth6_numb.y == "True" ,
                                                compl_mth6_cell.y == "True" ,
                                                compl_mth6_neur.y == "True" ,
                                                compl_mth6_weak.y == "True" ,
                                                compl_mth6_resp.y == "True" ,
                                                compl_mth6_othe.y == "True" ,
                                                compl_mth6_unkn.y == "True" ,na.rm=TRUE))

category <- "Other"
vprc_followed_up %>% filter(readm_mth6_any_spec.y == category | readm_mth6_any_spec.x ==
category) %>% select(readm_mth6_any_spec.y, readm_mth6_any_spec.x, id_number, pers_last_name.x)

# Demographic analysis
## Age
vprc_followed_up$age_in_yrs <- with(vprc_followed_up,as.numeric((as.Date(proc_date.x) -
as.Date(pers_dob.x))/ 365.25))
mean(vprc_followed_up$age_in_yrs)
sd(vprc_followed_up$age_in_yrs)

fup_timing_table <- as.data.frame(table(vprc_followed_up$flw_up_time))
colnames(fup_timing_table) <- c("Follow-up timing", "n")

print(xtable(fup_timing_table, caption="Patients by Follow-up Timing"), type="html",
include.rownames=FALSE, caption.placement = "top")

sex_table <- as.data.frame(table(vprc_followed_up$pers_sex.x))
colnames(sex_table) <- c("Sex", "n")

print(xtable(sex_table, caption="Patients by Gender"), type="html", include.rownames=FALSE,
caption.placement = "top")

number_compl<- as.data.frame(table(a$num_of_complications.y))
colnames(number_compl)<- c("Number of complications","Patients")
print(xtable(number_compl, caption="Number of Complications per Patient"), type="html",
include.rownames=FALSE, caption.placement = "top")

joint_table <- as.data.frame(table(vprc_followed_up$joint.x))
```

```
colnames(joint_table) <- c("Joint", "n")
print(xtable(joint_table, caption="Patients by Joint"), type="html", include.rownames=FALSE,
caption.placement = "top")
eval_flag <- TRUE

demographic_table <- vprc_followed_up %>% group_by(surgeon.x) %>% summarise(n=n(),
males=sum(pers_sex.x=="Male"), females=sum(pers_sex.x=="Female"),
mean_age=mean(age_in_yrs,na.rm=TRUE), sd_age=sd(age_in_yrs,na.rm=TRUE), hips=sum(joint.x=="Hip"),
knees=sum(joint.x=="Knee"),
flw_up_lt_6wks=sum(flw_up_time=="Less than 6 weeks", na.rm=TRUE),
flw_up_6_8wks=sum(flw_up_time=="6 to 8 weeks", na.rm=TRUE),
flw_up_3_5mths=sum(flw_up_time=="3 to 5 months", na.rm=TRUE),
flw_up_6mths=sum(flw_up_time=="6 months", na.rm=TRUE),
flw_up_7_12mths=sum(flw_up_time=="7 to 12 months", na.rm=TRUE))

demographic_table_all <- vprc_followed_up %>% summarise(n=n(), males=sum(pers_sex.x=="Male"),
females=sum(pers_sex.x=="Female"), mean_age=mean(age_in_yrs,na.rm=TRUE),
sd_age=sd(age_in_yrs,na.rm=TRUE), hips=sum(joint.x=="Hip"), knees=sum(joint.x=="Knee"),
flw_up_lt_6wks=sum(flw_up_time=="Less than 6 weeks", na.rm=TRUE),
flw_up_6_8wks=sum(flw_up_time=="6 to 8 weeks", na.rm=TRUE),
flw_up_3_5mths=sum(flw_up_time=="3 to 5 months", na.rm=TRUE),
flw_up_6mths=sum(flw_up_time=="6 months", na.rm=TRUE),
flw_up_7_12mths=sum(flw_up_time=="7 to 12 months", na.rm=TRUE))

demographic_table <- bind_rows(demographic_table, demographic_table_all)

dtable <- t(demographic_table)
colnames(dtable) <- dtable[1,]

demographic_table <- dtable[-1,]

kable(demographic_table)

# Calculating results
## Values calculated
get_kappa_results <- function(twocols, varname, kappa_results) {

  k2 <- irr::kappa2(twocols)
  k <- NULL
  try(k <- cohen.kappa(twocols), silent = TRUE)
  a <- agree(twocols)
  tbl <- table(twocols,exclude=NULL)
  k_df <- data.frame(varname=varname,
  k_subjects=k2$subjects,
  k_value=ifelse(!is.null(k),k$kappa,1),
  k2_value=k2$value,
  k2_p.value=k2$p.value,
  k_confid=ifelse(!is.null(k),paste("(",format(k$confid[[1]],digits=2),",
",format(k$confid[[5]],digits=2),")",sep=""),NA),
  a_method=a$method,
  a_subjects=a$subjects,
  a_raters=a$raters,
  a_irr.name=a$irr.name,
  a_value=a$value,
  sensitivity=ifelse(class(unlist(twocols[,2])) == "logical",
sensitivity(factor(unlist(twocols[,2])), factor(unlist(twocols[,1]))),
sensitivity(unlist(twocols[,2]), unlist(twocols[,1]))),
  specificity=ifelse(class(unlist(twocols[,2])) == "logical",
specificity(factor(unlist(twocols[,2])), factor(unlist(twocols[,1]))),
specificity(unlist(twocols[,2]), unlist(twocols[,1]))),
  ppv=ifelse(class(unlist(twocols[,2])) == "logical", posPredValue(factor(unlist(twocols[,2])),
factor(unlist(twocols[,1]))), posPredValue(unlist(twocols[,2]), unlist(twocols[,1]))),
  npv=ifelse(class(unlist(twocols[,2])) == "logical", negPredValue(factor(unlist(twocols[,2])),
factor(unlist(twocols[,1]))), negPredValue(unlist(twocols[,2]), unlist(twocols[,1]))),
  TT=tbl[1,1],
  TF=tbl[1,2],
  FF=tbl[2,2],
  FT=tbl[2,1]
  )
  if (is.null(kappa_results)) {
    kappa_results <- k_df
  } else {
    kappa_results <- rbind(kappa_results, k_df)
  }
  kappa_results[kappa_results$k_value==0,"k_confid"] <- NA
  kappa_results[is.nan(kappa_results$k2_p.value),"k2_p.value"] <- NA
  return(kappa_results)
}

calc_results <- function(df, df_thrombo, df_infection, df_joint, df_internal, df_subjective,
df_overall) {
  kappa_results <- NULL
```

```
  with(df, {
       kappa_results <- get_kappa_results(df[,c("readm_mth6.x", "readm_mth6.y")], "Readmission",
kappa_results)
       kappa_results <- get_kappa_results(df[,c("reop_mth6.x", "reop_mth6.y")], "Reoperation",
kappa_results)
       kappa_results <- get_kappa_results(df_thrombo[,c("compl_mth6_thromboembolic.x",
"compl_mth6_thromboembolic.y")], "Thromboembolic", kappa_results)
       kappa_results <- get_kappa_results(df[,c("compl_mth6_dvt_index.x",
"compl_mth6_dvt_index.y")], "DVT Index Leg", kappa_results)
       kappa_results <- get_kappa_results(df[,c("compl_mth6_dvt_other.x",
"compl_mth6_dvt_other.y")], "DVT Other Leg", kappa_results)
       kappa_results <- get_kappa_results(df[,c("compl_mth6_dvt_both.x",
"compl_mth6_dvt_both.y")],"DVT Both Legs", kappa_results)
       kappa_results <- get_kappa_results(df[,c("compl_mth6_pe.x", "compl_mth6_pe.y")], "Pulmonary
Embolism", kappa_results)
       kappa_results <- get_kappa_results(df_infection[,c("compl_mth6_infection.x",
"compl_mth6_infection.y")], "Infections", kappa_results)
       kappa_results <- get_kappa_results(df[,c("compl_mth6_ssi_oral_ab.x",
"compl_mth6_ssi_oral_ab.y")], "SSI requiring Oral AB", kappa_results)
       kappa_results <- get_kappa_results(df[,c("compl_mth6_ssi_iv_ab.x",
"compl_mth6_ssi_iv_ab.y")], "SSI requiring Intravenous AB", kappa_results)
       kappa_results <- get_kappa_results(df[,c("compl_mth6_cell.x", "compl_mth6_cell.y")],
"Cellulitis", kappa_results)
       kappa_results <- get_kappa_results(df_joint[,c("compl_mth6_joint.x", "compl_mth6_joint.y")],
"Joint Problems", kappa_results)
       kappa_results <- get_kappa_results(df[,c("compl_mth6_disl.x", "compl_mth6_disl.y")],
"Dislocation", kappa_results)
       kappa_results <- get_kappa_results(df[,c("compl_mth6_stif.x", "compl_mth6_stif.y")],
"Stiffness", kappa_results)
       kappa_results <- get_kappa_results(df[,c("compl_mth6_frac.x", "compl_mth6_frac.y")],
"Fracture", kappa_results)
       kappa_results <- get_kappa_results(df[,c("compl_mth6_legl.x", "compl_mth6_legl.y")], "Leg
Length Discrepency", kappa_results)
       kappa_results <- get_kappa_results(df[,c("compl_mth6_swel.x", "compl_mth6_swel.y")], "Joint
or Lower Leg Swelling", kappa_results)
       kappa_results <- get_kappa_results(df_internal[,c("compl_mth6_internal.x",
"compl_mth6_internal.y")], "Medical Complications", kappa_results)
       kappa_results <- get_kappa_results(df[,c("compl_mth6_resp.x", "compl_mth6_resp.y")],
"Respiratory Infection", kappa_results)
       kappa_results <- get_kappa_results(df[,c("compl_mth6_card.x", "compl_mth6_card.y")],
"Cardiac", kappa_results)
       kappa_results <- get_kappa_results(df[,c("compl_mth6_stro.x", "compl_mth6_stro.y")],
"Stroke", kappa_results)
       kappa_results <- get_kappa_results(df[,c("compl_mth6_blad.x", "compl_mth6_blad.y")], "Bladder
Infection/Retention", kappa_results)
       kappa_results <- get_kappa_results(df[,c("compl_mth6_neur.x", "compl_mth6_neur.y")],
"Neuropathy", kappa_results)
       kappa_results <- get_kappa_results(df_subjective[,c("compl_mth6_subjective.x",
"compl_mth6_subjective.y")], "Subjective Complications", kappa_results)
       kappa_results <- get_kappa_results(df[,c("compl_mth6_pain.x", "compl_mth6_pain.y")],
"Unexpected Pain", kappa_results)
       kappa_results <- get_kappa_results(df[,c("compl_mth6_numb.x", "compl_mth6_numb.y")],
"Parasthesia/Numbness", kappa_results)
       kappa_results <- get_kappa_results(df[,c("compl_mth6_weak.x", "compl_mth6_weak.y")], "Muscle
Weakness", kappa_results)
       kappa_results <- get_kappa_results(df[,c("compl_mth6_othe.x", "compl_mth6_othe.y")], "Other",
kappa_results)
       kappa_results <- get_kappa_results(df[,c("compl_mth6_unkn.x", "compl_mth6_unkn.y")],
"Unknown", kappa_results)
       kappa_results <- get_kappa_results(df_overall[,c("compl_mth6_overall.x",
"compl_mth6_overall.y")], "Overall Complications", kappa_results)

## Results in a table
       desired_cols <- c("varname", "k_subjects", "TT", "TF", "FT", "FF","sensitivity",
"specificity", "ppv", "npv", "a_value", "k_value")

      col_headings <- c("Variable", "No.of subjects", "TT", "TF", "FT", "FF", "Sensitivity",
"Specificity", "PPV", "NPV", "Agreement %", "kappa")
    kappa_results <- distinct(kappa_results)
    print(kable(kappa_results[,desired_cols],col.names=col_headings, digits=2))
  }
  )
}

calc_results(vprc_followed_up, vprc_followed_up_thromboembolic, vprc_followed_up_infection,
vprc_followed_up_joint, vprc_followed_up_internal, vprc_followed_up_subjective,
vprc_followed_up_overall)

# Results categorized by surgeons
# Surgeons’ names were used for original code but have been replaced to ensure confidentiality
calc_results(vprc_followed_up[vprc_followed_up$surgeon.x == "Surgeon
A",],vprc_followed_up_thromboembolic[vprc_followed_up_thromboembolic$surgeon.x == "Surgeon A",],
```

```
vprc_followed_up_infection[vprc_followed_up_infection$surgeon.x == "Surgeon A",],
vprc_followed_up_joint[vprc_followed_up_joint$surgeon.x == "Surgeon A",],
vprc_followed_up_internal[vprc_followed_up_internal$surgeon.x == "Surgeon
A",],vprc_followed_up_subjective[vprc_followed_up_subjective$surgeon.x == "Surgeon A",],
vprc_followed_up_overall[vprc_followed_up_overall$surgeon.x == "Surgeon A",])

calc_results(vprc_followed_up[vprc_followed_up$surgeon.x == "Surgeon
B",],vprc_followed_up_thromboembolic[vprc_followed_up_thromboembolic$surgeon.x == "Surgeon B",],
vprc_followed_up_infection[vprc_followed_up_infection$surgeon.x == "Surgeon B",],
vprc_followed_up_joint[vprc_followed_up_joint$surgeon.x == "Surgeon B",],
vprc_followed_up_internal[vprc_followed_up_internal$surgeon.x == "Surgeon
B",],vprc_followed_up_subjective[vprc_followed_up_subjective$surgeon.x == "Surgeon B",],
vprc_followed_up_overall[vprc_followed_up_overall$surgeon.x == "Surgeon B",])

calc_results(vprc_followed_up[vprc_followed_up$surgeon.x == "Surgeon
C",],vprc_followed_up_thromboembolic[vprc_followed_up_thromboembolic$surgeon.x == "Surgeon C",],
vprc_followed_up_infection[vprc_followed_up_infection$surgeon.x == "Surgeon C",],
vprc_followed_up_joint[vprc_followed_up_joint$surgeon.x == "Surgeon C",],
vprc_followed_up_internal[vprc_followed_up_internal$surgeon.x == "Surgeon
C",],vprc_followed_up_subjective[vprc_followed_up_subjective$surgeon.x == "Surgeon C",],
vprc_followed_up_overall[vprc_followed_up_overall$surgeon.x == "Surgeon C",])

calc_results(vprc_followed_up[vprc_followed_up$surgeon.x == "Surgeon
D",],vprc_followed_up_thromboembolic[vprc_followed_up_thromboembolic$surgeon.x == "Surgeon D",],
vprc_followed_up_infection[vprc_followed_up_infection$surgeon.x == "Surgeon D",],
vprc_followed_up_joint[vprc_followed_up_joint$surgeon.x == "Surgeon D",],
vprc_followed_up_internal[vprc_followed_up_internal$surgeon.x == "Surgeon
D",],vprc_followed_up_subjective[vprc_followed_up_subjective$surgeon.x == "Surgeon D",],
vprc_followed_up_overall[vprc_followed_up_overall$surgeon.x == "Surgeon D",])

calc_results(vprc_followed_up[vprc_followed_up$surgeon.x == "Surgeon
E",],vprc_followed_up_thromboembolic[vprc_followed_up_thromboembolic$surgeon.x == "Surgeon E",],
vprc_followed_up_infection[vprc_followed_up_infection$surgeon.x == "Surgeon E",],
vprc_followed_up_joint[vprc_followed_up_joint$surgeon.x == "Surgeon E",],
vprc_followed_up_internal[vprc_followed_up_internal$surgeon.x == "Surgeon
E",],vprc_followed_up_subjective[vprc_followed_up_subjective$surgeon.x == "Surgeon E",],
vprc_followed_up_overall[vprc_followed_up_overall$surgeon.x == "Surgeon E",])

calc_results(vprc_followed_up[vprc_followed_up$surgeon.x == "Surgeon
F",],vprc_followed_up_thromboembolic[vprc_followed_up_thromboembolic$surgeon.x == "Surgeon F",],
vprc_followed_up_infection[vprc_followed_up_infection$surgeon.x == "Surgeon F",],
vprc_followed_up_joint[vprc_followed_up_joint$surgeon.x == "Surgeon F",],
vprc_followed_up_internal[vprc_followed_up_internal$surgeon.x == "Surgeon
F",],vprc_followed_up_subjective[vprc_followed_up_subjective$surgeon.x == "Surgeon F",],
vprc_followed_up_overall[vprc_followed_up_overall$surgeon.x == "Surgeon F",])

# Results categorized by joint

calc_results(vprc_followed_up[vprc_followed_up$joint.x ==
"Hip",],vprc_followed_up_thromboembolic[vprc_followed_up_thromboembolic$joint.x == "Hip",],
vprc_followed_up_infection[vprc_followed_up_infection$joint.x == "Hip",],
vprc_followed_up_joint[vprc_followed_up_joint$joint.x == "Hip",],
vprc_followed_up_internal[vprc_followed_up_internal$joint.x ==
"Hip",],vprc_followed_up_subjective[vprc_followed_up_subjective$joint.x == "Hip",],
vprc_followed_up_overall[vprc_followed_up_overall$joint.x == "Hip",])

calc_results(vprc_followed_up[vprc_followed_up$joint.x ==
"Knee",],vprc_followed_up_thromboembolic[vprc_followed_up_thromboembolic$joint.x == "Knee",],
vprc_followed_up_infection[vprc_followed_up_infection$joint.x == "Knee",],
vprc_followed_up_joint[vprc_followed_up_joint$joint.x == "Knee",],
vprc_followed_up_internal[vprc_followed_up_internal$joint.x ==
"Knee",],vprc_followed_up_subjective[vprc_followed_up_subjective$joint.x == "Knee",],
vprc_followed_up_overall[vprc_followed_up_overall$joint.x == "Knee",])

# Results categorized by follow-up time

calc_results(vprc_followed_up[vprc_followed_up$flw_up_time == "Less than 6
weeks",],vprc_followed_up_thromboembolic[vprc_followed_up_thromboembolic$flw_up_time == "Less than 6
weeks",], vprc_followed_up_infection[vprc_followed_up_infection$flw_up_time == "Less than 6
weeks",], vprc_followed_up_joint[vprc_followed_up_joint$flw_up_time == "Less than 6 weeks",],
vprc_followed_up_internal[vprc_followed_up_internal$flw_up_time == "Less than 6
weeks",],vprc_followed_up_subjective[vprc_followed_up_subjective$flw_up_time == "Less than 6
weeks",], vprc_followed_up_overall[vprc_followed_up_overall$flw_up_time == "Less than 6 weeks",])

calc_results(vprc_followed_up[vprc_followed_up$flw_up_time == "6 to 8
weeks",],vprc_followed_up_thromboembolic[vprc_followed_up_thromboembolic$flw_up_time == "6 to 8
weeks",], vprc_followed_up_infection[vprc_followed_up_infection$flw_up_time == "6 to 8 weeks",],
vprc_followed_up_joint[vprc_followed_up_joint$flw_up_time == "6 to 8 weeks",],
vprc_followed_up_internal[vprc_followed_up_internal$flw_up_time == "6 to 8
weeks",],vprc_followed_up_subjective[vprc_followed_up_subjective$flw_up_time == "6 to 8 weeks",],
vprc_followed_up_overall[vprc_followed_up_overall$flw_up_time == "6 to 8 weeks",])
```

```
calc_results(vprc_followed_up[vprc_followed_up$flw_up_time == "3 to 5
months",],vprc_followed_up_thromboembolic[vprc_followed_up_thromboembolic$flw_up_time == "3 to 5
months",], vprc_followed_up_infection[vprc_followed_up_infection$flw_up_time == "3 to 5 months",],
vprc_followed_up_joint[vprc_followed_up_joint$flw_up_time == "3 to 5 months",],
vprc_followed_up_internal[vprc_followed_up_internal$flw_up_time == "3 to 5
months",],vprc_followed_up_subjective[vprc_followed_up_subjective$flw_up_time == "3 to 5 months",],
vprc_followed_up_overall[vprc_followed_up_overall$flw_up_time == "3 to 5 months",])

calc_results(vprc_followed_up[vprc_followed_up$flw_up_time == "6
months",],vprc_followed_up_thromboembolic[vprc_followed_up_thromboembolic$flw_up_time == "6
months",], vprc_followed_up_infection[vprc_followed_up_infection$flw_up_time == "6 months",],
vprc_followed_up_joint[vprc_followed_up_joint$flw_up_time == "6 months",],
vprc_followed_up_internal[vprc_followed_up_internal$flw_up_time == "6
months",],vprc_followed_up_subjective[vprc_followed_up_subjective$flw_up_time == "6 months",],
vprc_followed_up_overall[vprc_followed_up_overall$flw_up_time == "6 months",])

calc_results(vprc_followed_up[vprc_followed_up$flw_up_time == "7 to 12
months",],vprc_followed_up_thromboembolic[vprc_followed_up_thromboembolic$flw_up_time == "7 to 12
months",], vprc_followed_up_infection[vprc_followed_up_infection$flw_up_time == "7 to 12 months",],
vprc_followed_up_joint[vprc_followed_up_joint$flw_up_time == "7 to 12 months",],
vprc_followed_up_internal[vprc_followed_up_internal$flw_up_time == "7 to 12
months",],vprc_followed_up_subjective[vprc_followed_up_subjective$flw_up_time == "7 to 12 months",],
vprc_followed_up_overall[vprc_followed_up_overall$flw_up_time == "7 to 12 months",])
```
